# *Giardia intestinalis* thymidine kinase is a high-affinity enzyme crucial for DNA synthesis and an exploitable target for drug discovery

**DOI:** 10.1016/j.jbc.2022.102028

**Published:** 2022-05-11

**Authors:** Sascha Krakovka, Farahnaz Ranjbarian, Lucas A. Luján, Alicia Saura, Nicolai B. Larsen, Alejandro Jiménez-González, Anna Reggenti, Hugo D. Luján, Staffan G. Svärd, Anders Hofer

**Affiliations:** 1 Department of Cell and Molecular Biology, BMC, Uppsala University, Uppsala, Sweden; 2 Department of Medical Biochemistry and Biophysics, Umeå University, Umeå, Sweden; 3 Centro de Investigación y Desarrollo en Immunología y Enfermedades Infecciosas (CIDIE), Consejo Nacional de Investigaciones Científicas y Técnicas (CONICET)/Universidad Católica de Córdoba (UCC), Cordoba, Argentina; 4 Statens Serum Institut, København S, Denmark

**Keywords:** thymidine kinase, deoxyribonucleoside kinase, deoxynucleoside kinase, deoxyribonucleoside salvage, deoxynucleoside salvage, *Giardia intestinalis*, *Giardia lamblia*, *Giardia duodenum*, azidothymidine, zidovudine, AZT, azidothymidine, DMSO, dimethyl sulfoxide, FDA, fluorescein diacetate, PI, propidium iodide, TCA, trichloroacetic acid, TEV, tobacco etch virus

## Abstract

Giardiasis is a diarrheal disease caused by the unicellular parasite *Giardia intestinalis*, for which metronidazole is the main treatment option. The parasite is dependent on exogenous deoxyribonucleosides for DNA replication and thus is also potentially vulnerable to deoxyribonucleoside analogs. Here, we characterized the *G. intestinalis* thymidine kinase, a divergent member of the thymidine kinase 1 family that consists of two weakly homologous parts within one polypeptide. We found that the recombinantly expressed enzyme is monomeric, with 100-fold higher catalytic efficiency for thymidine compared to its second-best substrate, deoxyuridine, and is furthermore subject to feedback inhibition by dTTP. This efficient substrate discrimination is in line with the lack of thymidylate synthase and dUTPase in the parasite, which makes deoxy-UMP a dead-end product that is potentially harmful if converted to deoxy-UTP. We also found that the antiretroviral drug azidothymidine (AZT) was an equally good substrate as thymidine and was active against WT as well as metronidazole-resistant *G. intestinalis* trophozoites. This drug inhibited DNA synthesis in the parasite and efficiently decreased cyst production *in vitro*, which suggests that it could reduce infectivity. AZT also showed a good effect in *G. intestinalis*–infected gerbils, reducing both the number of trophozoites in the small intestine and the number of viable cysts in the stool. Taken together, these results suggest that the absolute dependency of the parasite on thymidine kinase for its DNA synthesis can be exploited by AZT, which has promise as a future medication effective against metronidazole-refractory giardiasis.

*Giardia intestinalis* (also known as *Giardia duodenalis* and *Giardia lamblia*) causes giardiasis and is one of the major parasitic causes of diarrhea with 190 million symptomatic infections per year worldwide, adding up to ∼171,100 daily adjusted life years lost (1). *G. intestinalis* is a noninvasive, unicellular, and binuclear parasite, and it is spread as infectious cysts from drinking water and contaminated surfaces. After passage through the stomach, the parasites excyst, forming the trophozoite form that attaches to the upper small intestine and starts multiplying. Some of the cells detach and move to the lower intestines, where a stress response induces encystation. During encystation, the genome is duplicated twice, leading to the cell acquiring a total of four nuclei and 16 genome copies. The resulting cystic form has low metabolic activity and can withstand diverse environmental factors like UV light, low or high salt concentrations, and low temperatures. It is thereby adapted to spreading between hosts (2). The symptoms of giardiasis vary from asymptomatic infections to severe diarrhea and abdominal cramping. In most cases, the immune system clears the parasites after 2 to 4 weeks, but sometimes the infection becomes chronic with long-term effects such as food allergies, chronic fatigue syndrome, and irritable bowel syndrome. These effects can also occur in patients without detectable infection and may persist for over a decade after the initial disease (2, 3). Also, several recent studies have shown a connection between *G. intestinalis* infections and delayed growth of children in middle- and low-income countries (4, 5, 6). Both the acute symptoms as well as the long-term effects mandate rapid treatment of the disease.

There is currently no human vaccine providing protection from *G. intestinalis*, and chemotherapy with metronidazole and other 5-nitroimidazoles is the major treatment option. The 5-nitroimidazoles specifically target microaerophilic and anaerobic cells because the active forms of these drugs are rapidly inactivated in oxygen-rich environments (7). The major 5-nitroimidazole used is metronidazole, which has many side effects, including nausea, vomiting, metallic taste, and headaches, and long-term use is associated with neurological symptoms and reduced liver function (8, 9). In recent years, *G. intestinalis* drug resistance rates of 20 to 40% have been reported in the clinic, making the search for an alternative to metronidazole all the more urgent (10, 11, 12). Crossresistance to other 5-nitroimidazoles is also common and is easily inducible in the laboratory (13, 14). This highlights the importance of finding new classes of molecules with different modes of action to treat giardiasis.

The parasitic lifestyle has the advantage that a number of essential metabolites are already present in the host, and consequently parasites often lack metabolic genes that are needed in free-living organisms (15). DNA biosynthesis requires the four deoxyribonucleoside triphosphates (dNTPs) dCTP, dTTP, dATP, and dGTP, but *G. intestinalis* has neither ribonucleotide reductase nor thymidylate synthase (Fig. 1), which are key enzymes for *de novo* synthesis of dNTPs and dTTP, respectively (16, 17, 18). The lack of these two enzymes makes the parasite dependent on salvaging deoxyribonucleosides from the host, and this process requires membrane transporters and kinases for the uptake and phosphorylation of the deoxyribonucleosides (19). The addition of the first phosphate is generally rate limiting and is catalyzed by deoxyribonucleoside kinases (19). The complete dependency on deoxyribonucleoside salvage might be exploited for drug development in two different ways, either by blocking one of the salvage enzymes or by using substrate analogs that upon phosphorylation mimic the natural dNTPs and act as chain terminators in DNA synthesis. The dependency of the organism on the deoxyribonucleoside kinases for normal DNA replication precludes the ability to acquire drug resistance by downregulating the enzymes.

**Figure 1:**
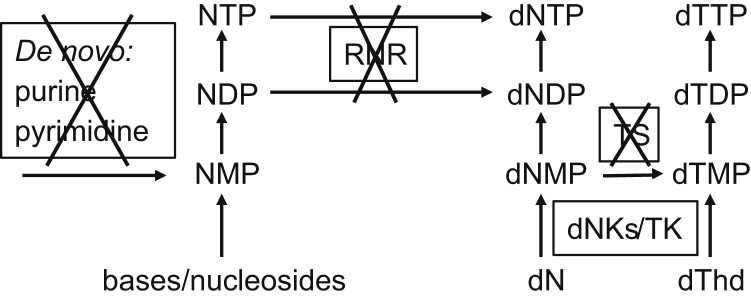
**Nucleotide metabolism in *G. intestinalis*.** The parasite lacks many pathways in nucleotide metabolism including ribonucleotide reductase (RNR) and thymidylate synthase (TS), which are crucial for general dNTP synthesis and dTTP synthesis, respectively. Consequently, the parasite is completely dependent on deoxyribonucleoside kinases to make dNTPs. There are three such kinases in the *G. intestinalis* WB genome, including two deoxyribonucleoside kinases from the dCK-dGK-TK2 family (dNKs, ORFs 4558 and 17451) and one from the thymidine kinase 1 family (TK, ORF 8364).

Two deoxyribonucleoside kinase activities were previously purified and characterized from *G. intestinalis* (20), with one of them phosphorylating purines and the other pyrimidines. However, at that time the corresponding genes were not known and subsequent genomic analyses showed that *G. intestinalis* has three deoxyribonucleoside genes, indicating that the initial studies either missed one of the kinases or that two of them were copurified. One of the *G. intestinalis* deoxyribonucleoside kinase genes is from the same family as human thymidine kinase 1 (TK1). The members of this family typically have a restrictive base specificity only recognizing thymidine and deoxyuridine among naturally occurring substrates. The other two *G. intestinalis* genes are from the second deoxyribonucleoside kinase family, which includes three human enzymes: deoxycytidine kinase, deoxyguanosine kinase, and thymidine kinase 2. This family has a broader substrate specificity and includes members that phosphorylate purines as well as pyrimidines. One of the incentives behind the current study was to investigate whether the *G. intestinalis* thymidine kinase has a strict base specificity similar to other members of the TK1 family or if this could be the same enzyme as in the fraction that phosphorylated both thymidine and deoxycytidine in the initial study (20). Interestingly, we found that the *G. intestinalis* thymidine kinase has the most restrictive base specificity ever reported for any enzyme in the TK1 family. It had a 100 times higher catalytic efficiency with thymidine than to the second-best natural substrate, deoxyuridine, which could be a way for the parasite to prevent deoxyuridine triphosphate (dUTP) production and thus limit the consequent misincorporation of this nucleotide into DNA. We observed no detectable activity with deoxycytidine. In contrast to the strict base specificity, the recognition of the sugar moiety was more relaxed and azidothymidine (AZT) was an efficient substrate for inhibiting parasite DNA replication, *in-vitro* cell proliferation, and parasite burden in *G. intestinalis*–infected rodents. We also found encystation to be efficiently hindered by both AZT and metronidazole but with AZT also affecting the DNA content in each cyst. Finally, cell lines resistant to metronidazole and related compounds (21) showed no better tolerance toward AZT than metronidazole-susceptible cell lines.

All together, we show in this work that AZT appears to be a good drug candidate for the treatment of giardiasis by exploiting an essential metabolic pathway in *G. intestinalis*. Moreover, it has a clear mechanism of action (inhibition of DNA replication) affecting cell proliferation as well as encystation and does not show crossresistance with the main class of drugs used today.

## Results

### General description of the *G. intestinalis* thymidine kinase

**Figure 2:**
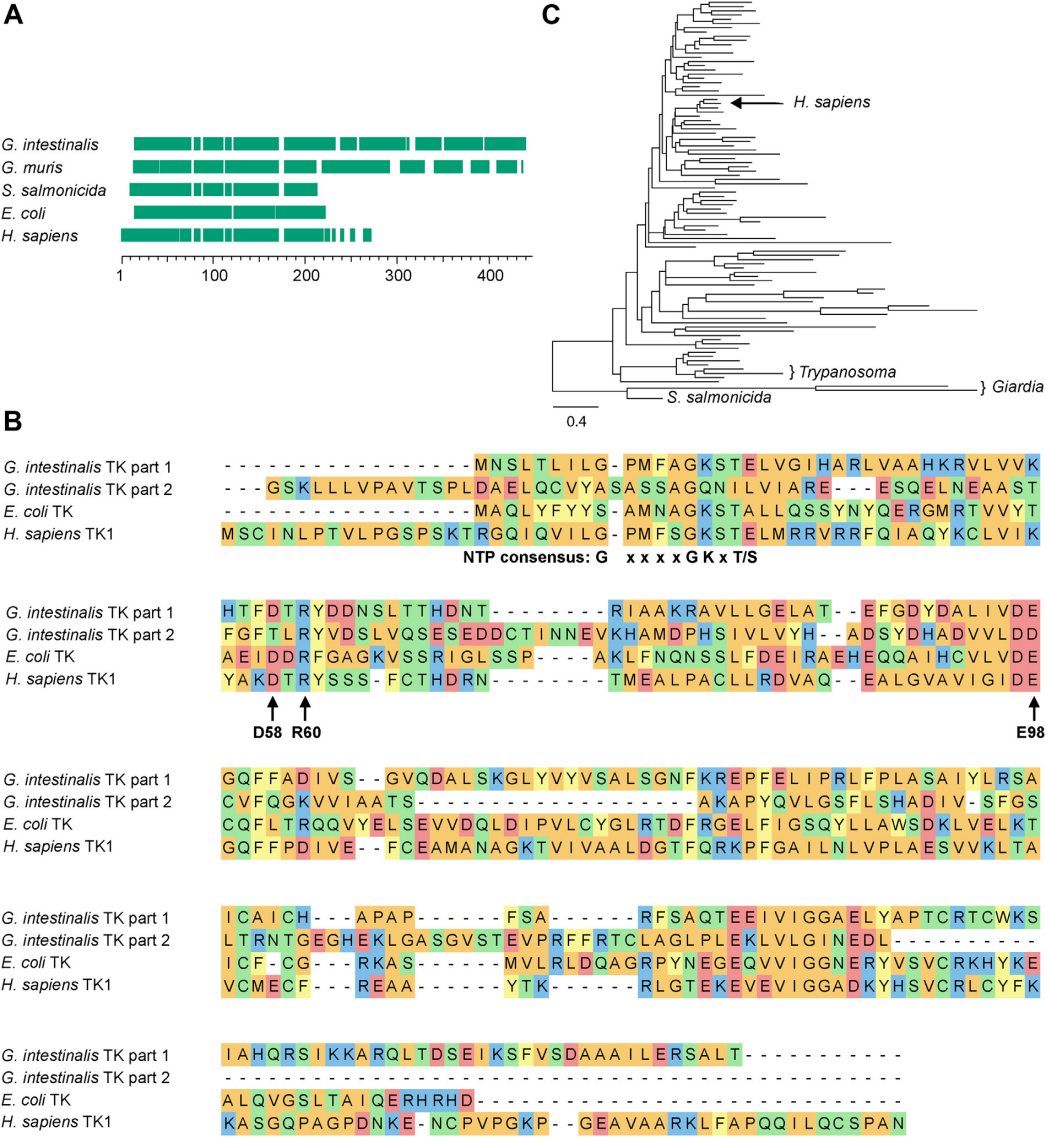
**Amino acid sequence alignment of thymidine kinases from the TK1 family.***A*, alignment overview of TK1 family members including thymidine kinases from two *Giardia* species and the related parasite *Spironucleus salmonicida*. *Green blocks* represent the aligned parts with spaces showing gaps in the alignment. *B*, detailed view showing the amino acid sequences from a second alignment performed with three of the thymidine kinases. The *G. intestinalis* thymidine kinase sequence was split prior to the alignment into part 1 (amino acids 1–210) and part 2 (amino acids 211–392). The alignments performed in (*A*) and (*B*) were generated with Clustal Omega, and the color code in (*B*) indicates positive charge (*blue*), negative charge (*red*), nonpolar residues (*orange*), polar residues (*green*), and aromatic residues (*yellow*). The highlighted residues are the NTP consensus sequence needed for ATP binding, D58, which is needed for interaction with the 3′position of the deoxyribose, R60, which is involved in the formation of the transition state, and E98, which is needed for abstraction of the hydrogen from the 5′ end of the deoxyribose in order to initiate the enzymatic reaction. All of the highlighted residues were present in the first part of the *G. intestinalis* thymidine kinase, while many of them were lacking in the second part. *C*, phylogenetic tree of the eukaryotic clade of the TK1 family. For a more detailed view of the phylogenetic tree and a description of the parameters used to construct the tree, see Fig. S2.

The *G. intestinalis* thymidine kinase has a theoretical molecular mass of 42 kDa (gene GL50803_8364 in the *G. intestinalis* WB strain), which is nearly double the size compared to the corresponding mammalian (TK1) and *Escherichia coli* enzymes (Fig. 2*A*). The large size is comparable to the corresponding enzyme from *Trypanosoma brucei*, which is composed of two consecutive thymidine kinase sequences in the same polypeptide but with the first one being inactive due to the lack of some important residues (22). The full-length *T. brucei* sequence failed to align properly with the other sequences and is not included in Figure 2*A*. The enzyme from *G. intestinalis* was also found to consist of two parts, but in this case, the first part seemed to be the active kinase (Fig. 2*B*). This part is 41% identical to human thymidine kinase (TK1) and contains the important residues needed for enzyme activity (Fig. 2*B*). The second part has only weak homology with other thymidine kinases and lacks many important residues (Fig. 2*B*). The murine parasite *Giardia muris* has the same setup with two consecutive TK1 domains and a more conserved N-terminal part, whereas the related salmon parasite *Spironucleus salmonicida* thymidine kinase is shorter with only one TK1 domain (Figs. 2*A* and S1). Studies from *T. brucei* showed that the purpose of the inactive domain is to stabilize the protein and to help increase the affinity of the substrate for the active domain (22). In *G. intestinalis*, the homology between the two domains is much lower (13% identity), and it is therefore impossible to be sure if the second part originated from a thymidine kinase. BLAST and domain searches did not pick up any other homologous genes or domains. A phylogenetic analysis of *G. intestinalis* thymidine kinase (Figs. 2*C* and S2) showed that all diplomonad TK homologs are grouped together in a very divergent cluster compared with the rest of the eukaryotic taxa. The tree topology and the branch support values also excluded the possibility of a horizontal gene transfer from the host (98.7/100 and 97.3/99, respectively), and this is an advantage for developing drugs that specifically target the pathogen.

### Cloning, expression, and general characterization of the *G. intestinalis* thymidine kinase

**Figure 3:**
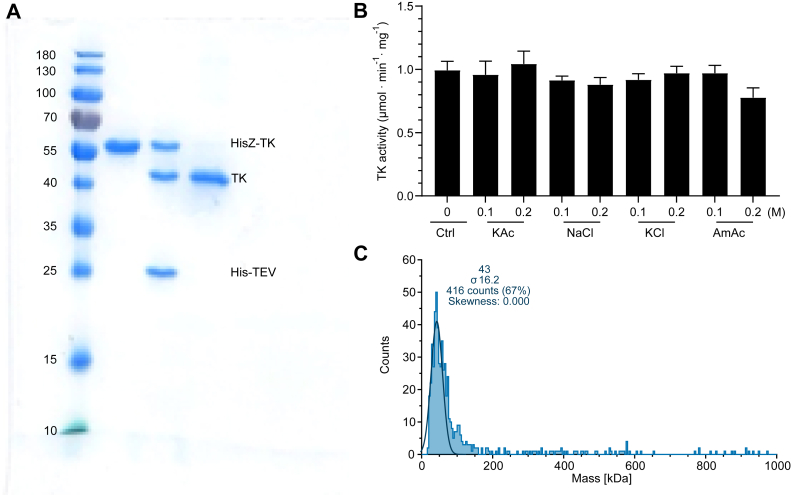
**Purification and general characterization of the recombinantly expressed *G. intestinalis* thymidine kinase.***A*, SDS-PAGE showing the purified fusion construct of His-tagged protein Z attached to the thymidine kinase (HisZ-TK). *Lane 1* shows the full-length construct after nickel agarose chromatography (theoretically 52 kDa), *lane 2* shows the same protein after cleavage, and *lane 3* shows the purified nontagged thymidine kinase (TK, theoretically 42 kDa). The final nontagged protein was collected in the flow-through after cleavage and repurification on nickel agarose to remove noncleaved protein, HisZ (10 kDa, not visible in the gel), and His-tagged TEV protease (27 kDa). *B*, the effect of different salts on thymidine kinase enzyme activity. Standard enzyme assay conditions were used in the assays, and the thymidine concentration was 0.2 mM. The results in (*B*) represent the average of three independent experiments with standard errors indicated. *C*, mass photometry analysis of *G. intestinalis* thymidine kinase showed that it is a monomer. The measured molecular mass was close to the theoretical mass of 42 kDa. TEV, tobacco etch virus.

The *G. intestinalis* thymidine kinase gene was cloned into a pET-Z vector for expression in *E. coli* as a fusion construct with a cleavable His_6_-tagged protein Z attached to the thymidine kinase. Figure 3*A* shows an SDS gel from different steps during the purification process yielding a homogeneous nontagged protein in the final step. General characterization of the purified thymidine kinase showed that the enzyme activity was rather insensitive to different salts tested up to 0.2 M (Fig. 3*B*) and that DTT had a slightly stimulatory effect (Fig. S3). The enzyme activity was linear with respect to time and enzyme concentration (Fig. S4).

Mass photometry analysis showed that the *G. intestinalis* thymidine kinase is a monomer (Fig. 3*C*). This is similar to the *T. brucei* thymidine kinase, which is also monomeric (22), but different from the mammalian enzyme (TK1) that is in a dimer–tetramer equilibrium (23). These results can be explained by the polypeptide structures where both the *G. intestinalis* and *T. brucei* enzymes are much larger than the human enzyme and can be viewed as pseudodimers, although the active part of the enzyme is in opposite orientations in the two cases.

### Phosphate donor specificity of the *G. intestinalis* thymidine kinase

The *G. intestinalis* thymidine kinase had a relaxed phosphate donor specificity giving high activities with all NTPs (Table 1). ATP and GTP were the best phosphate donors as judged by their catalytic efficiencies (*k*_cat_/*K*_*m*_), which were approximately the double as compared to the corresponding values with the pyrimidine NTPs. Although ATP had a significantly lower *k*_cat_ than the other NTPs, this was compensated for by a low *K*_*m*_ resulting in the high catalytic efficiency. The remaining experiments were performed with ATP, which has both the lowest *K*_*m*_ value and is generally the most abundant nucleotide in cells and is therefore likely to bind the enzyme most often of the four NTPs under physiological conditions.

**Table 1:** Kinetic parameters of the *G. intestinalis* thymidine kinase with different phosphate donors and substrates

NTP	*K*_*m*_ (μM)	*V*_max_/mg (μmol/min)	*k*_cat_ (s^−1^)	*k*_cat_/*K*_*m*_ (M^−1^ s^−1^)
ATP	467 ± 29	0.93 ± 0.10	0.65	1.4 × 10^3^
GTP	1303 ± 78	2.41 ± 0.11	1.69	1.3 × 10^3^
UTP	1927 ± 269	1.66 ± 0.21	1.16	6.0 × 10^2^
CTP	2396 ± 199	1.70 ± 0.12	1.19	5.0 × 10^2^

Substrate	*K*_*m*_ (μM)	*V*_max_/mg (μmol/min)	*k*_cat_ (s^−1^)	*k*_cat_/*K*_*m*_ (M^−1^ s^−1^)

Thd	0.069 ± 0.007	0.98 ± 0.09	0.67	1.00 × 10^7^
dUrd	5.3 ± 1.3	1.02 ± 0.06	0.67	1.34 × 10^5^
dCyd		<0.005^a^		
dAdo		<0.005^a^		
dGuo		<0.01^a^		
AZT	0.044 ± 0.002 (12%)	0.64 ± 0.03	0.60	1.00 × 10^7^
D4T	39 ± 13	0.25 ± 0.01	0.17	4.5 × 10^3^
AraT	425 ± 47	0.68 ± 0.16	0.48	1.1 × 10^3^

The phosphate donor experiments were performed with 1 μM thymidine as the substrate. The numbers represent the average of at least three independent experiments with standard errors indicated.

a Nondetectable. The measurements were performed with 200 μM substrate and 100 ng enzyme.

### Substrate specificity of the *G. intestinalis* thymidine kinase

**Figure 4:**
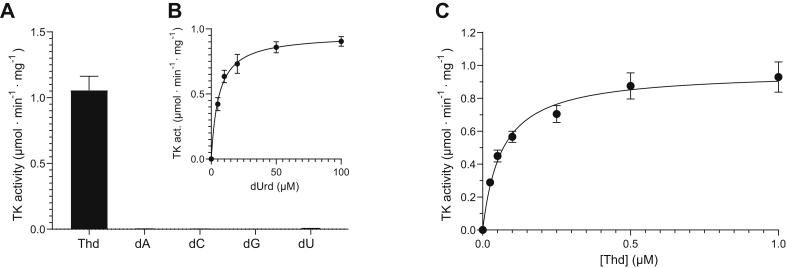
**Substrate specificity of the *G. intestinalis* thymidine kinase.***A*, a multisubstrate enzyme assay showed that thymidine was the main substrate for the *G. intestinalis* thymidine kinase. The enzyme activities with the other deoxyribonucleosides in this context in which they need to compete with thymidine were close to the detection limit with deoxyuridine being the second-best substrate (measured activity: 0.0047 ± 0.0019 μmol·min^−1^ mg^−1^). *B* and *C*, the enzyme activity as a function of the concentration of deoxyuridine and thymidine when tested separately. The curves in (*B* and *C*) were fitted by nonlinear regression to the Michaelis–Menten function using the GraphPad Prism 9.3.1 software (R^2^ = 0.89 for dUrd and R^2^ = 0.81 for Thd). The graphs in (*A*–*C*) represent the average of at least three independent experiments with standard errors indicated.

The previous finding that a protein fraction purified from *G. intestinalis* cells could phosphorylate both thymidine and deoxycytidine (20) inspired us to test the thymidine kinase substrate specificity in an assay where all main cellular deoxyribonucleosides are present simultaneously. Thymidine was by far the best substrate, and the enzyme activities with most other substrates were negligible (Fig. 4*A*). The low activity with the second-best substrate, deoxyuridine, caught our interest, and further studies showed that the *V*_max_ value was similar to that for thymidine but that the *K*_*m*_ values for the two substrates differed by 100-fold, indicating a much lower affinity for deoxyuridine (Table 1). This was also evident by the large difference in concentration needed to reach a given activity (Fig. 4, *B* and *C*).

Table 1 summarizes the results from a more extensive analysis of the substrate specificity of the *G. intestinalis* thymidine kinase. The base specificity was very rigid giving a much higher catalytic efficiency (*k*_cat_/*K*_*m*_) for thymidine when compared to deoxyuridine and no detectable activity with deoxycytidine, deoxyadenosine, or deoxyguanosine. In contrast, greater flexibility was allowed for the sugar moiety (at least in the 3′ position), and AZT was an excellent substrate having an equivalent catalytic efficiency as thymidine. The 2′ position of the sugar was more critical, and Ara-T and D4T were both poor substrates giving 3 to 4 orders of magnitude lower catalytic efficiency than with AZT.

### *G. intestinalis* thymidine kinase activity regulation

**Figure 5:**
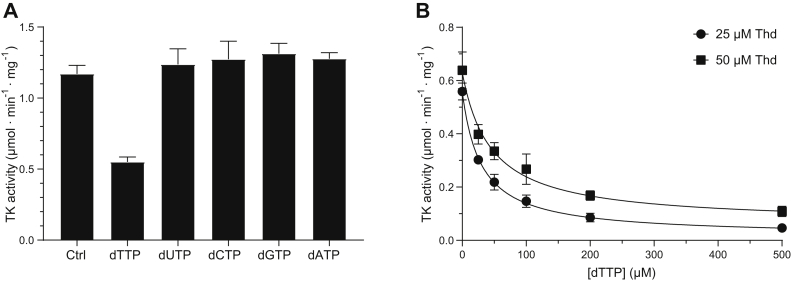
**Inhibition of the *G. intestinalis* thymidine kinase activity with dTTP.***A*, enzyme activity measured with 200 μM thymidine as the substrate in the presence of different dNTPs at 200 μM. *B*, enzyme activity with 25 μM and 50 μM thymidine and variable concentrations of dTTP as the inhibitor. The curves in (*B*) were fitted by nonlinear regression to the inhibitor *versus* response (three parameters) function in GraphPad Prism 9.3.1 (IC_50_ = 30 ± 3 μM and R^2^ = 0.99 for the 25 μM series; IC_50_ = 43 ± 1 μM and R^2^ = 0.96 for the 50 μM series). The graphs in (*A*) and (*B*) represent the average of three independent experiments with standard errors, except for the 50 μM series in (*B*) (n = 2).

Deoxyribonucleoside kinases are typically regulated in a nonconventional way that does not require an allosteric site. Instead, the dNTP regulator binds with its nucleobase attached to the substrate site and its phosphate groups entering into the region where the phosphates of ATP normally bind (19, 24). In this way, the regulator can compete for both ATP and the deoxyribonucleoside and thereby inhibit the enzyme. Our studies of the *G. intestinalis* thymidine kinase indicated a similar type of regulation, where dTTP was the only dNTP that seemed to inhibit the enzyme (Fig. 5*A*). The inhibition was modest using this experimental setup with only a 50% reduction in enzyme activity despite the high concentration of dTTP (200 μM). As expected with the established inhibition model, a much stronger inhibition could be obtained with lower concentrations of thymidine, supporting the hypothesis that the regulator and substrate compete for the same binding pocket (Fig. 5*B*). The dTTP concentration required to cause 50% inhibition was roughly equal to the substrate concentration in the two series shown in Figure 5*B*, indicating similar binding strengths for the substrate and the regulator.

### Antigiardial activity of AZT on WT and metronidazole-resistant strains

A proliferation assay with the WB strain of *G. intestinalis* showed that AZT was nearly as efficient as metronidazole and that the inhibitory effect was independent of whether the cells were resistant to metronidazole or not (Table 2 and Fig. S5). In contrast, the two resistant cell lines (M1 and M2) showed clearly elevated EC_50_ levels in the control experiments with metronidazole. Also included in Table 2 is an M1-derived revertant that has lost metronidazole resistance (M1NR). This cell line was as sensitive to both drugs as the WB parent strain. These general conclusions were similar regardless of the incubation time with the drug. The main difference between the 48 h and 72 h time series in Table 2 is that the general sensitivity to the drugs increased when the incubation was longer. The M1 and M2 cell lines used in this study are not only resistant to metronidazole but also against other related antigiardial compounds including tinidazole, ornidazol and nitazoxanide (21).

**Table 2:** EC_50_ values in μM of metronidazole and AZT against *G. intestinalis* using WT (WB), metronidazole-resistant (M1 and M2), and resistance-revertant (M1NR) cell lines

	WB	M1	M2	M1NR
**72 h incubation**				
AZT	4.38 ± 0.16	3.55 ± 0.13	1.63 ± 0.17	4.58 ± 0.34
Metronidazole	2.41 ± 0.24 (4.6%)	13.7 ± 1.5	9.79 ± 0.24	1.94 ± 0.28
**48 h incubation**				
AZT	6.0 ± 1.8	8.40 ± 0.23	3.9 ± 1.6	6.52 ± 0.87
Metronidazole	5.36 ± 0.27 (10%)	30.9 ± 4.6	14.3 ± 3.2	3.70 ± 0.21

The numbers represent the average of three independent experiments with standard errors indicated.

### Effect of AZT on *G. intestinalis* DNA synthesis

**Figure 6:**
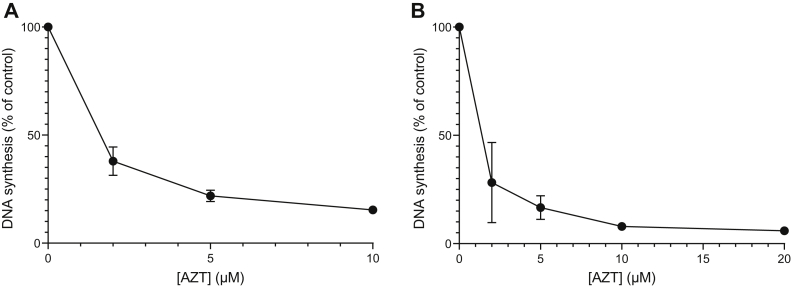
**Inhibition of *G. intestinalis* DNA synthesis with different concentration of AZT.***A*, the parasites were incubated with 10.5 nM ^3^H-dCyd and various concentrations of AZT for 1 h under normal growth conditions. *B*, similar to (*A*) but with 13 nM ^3^H-dAdo as the radioactive tracer and including 2 μM deoxycoformycin to protect dAdo from deamination. The recorded radioactivity in cellular DNA is shown as the percentage compared to the activity in cells treated without AZT. The results are based on three and two independent experiments for dCyd and dAdo, respectively, with standard errors indicated. AZT, azidothymidine.

AZT is best known for its ability to block HIV reverse transcriptase, but it also inhibits mammalian DNA synthesis when used at a higher dose. To test the effect of AZT on *G. intestinalis* DNA synthesis, parasites were incubated with ^3^H-labeled deoxycytidine or deoxyadenosine and different concentrations of AZT for 1 h. The radioactivity in DNA is an indicator of the replication rate in this kind of experiment. As shown in Figure 6, AZT had a strong impact on DNA synthesis with more than 50% inhibition already at 2 μM. This concentration is in a similar range as the EC_50_ determinations of *G. intestinalis* proliferation (Table 2), supporting the observation that the inhibition of DNA synthesis is the major effect of the drug. Figure 6 also shows that the results are similar regardless of the radioactive tracer used (deoxycytidine or deoxyadenosine), which strengthens the conclusion that AZT inhibits DNA synthesis rather than affecting deoxyribonucleoside uptake or metabolism.

### Efficacy of compounds against encystation

**Figure 7:**
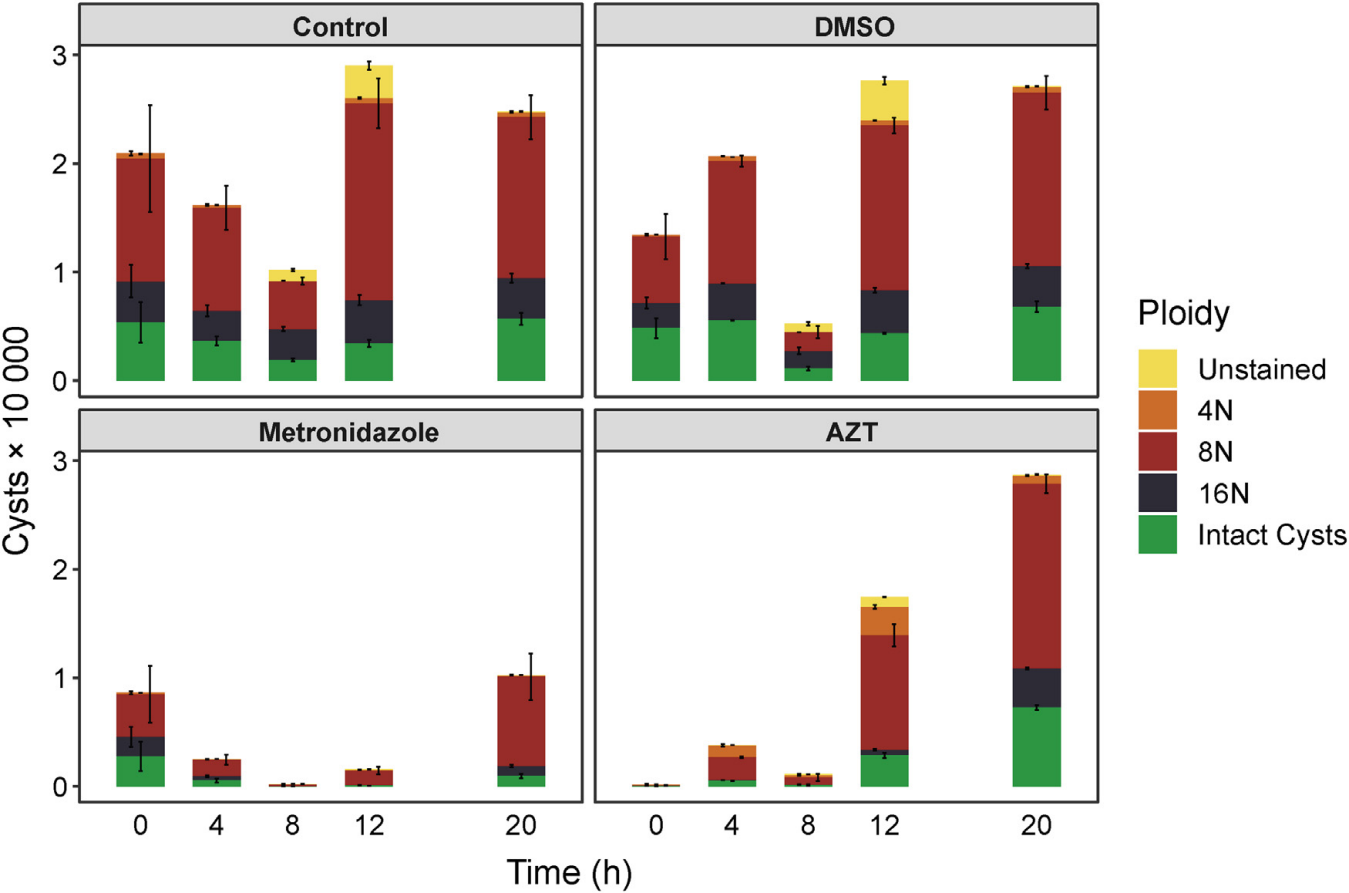
**The effect of AZT and metronidazole on *G. intestinalis* cyst formation and total cell numbers.** Treatment with pure encystation medium (control), DMSO, AZT, or metronidazole started at different time points during the encystation process. After completion of the encystation period (28 h) and subsequent treatment, the cells were categorized based on their staining with FDA (measuring metabolic activity) and PI (measuring DNA content). The PI staining requires a leaky membrane, and different genome amplification levels could therefore not be determined in the intact FDA^+^ cysts. Numbers given are normalized to be per 100,000 seeded trophozoites. Intact cysts (FDA^+^, PI^−^) are colored *green*, whereas the PI^+^ cysts (nearly all of them FDA^−^) are categorized according to their ploidy into 4N (*orange*), 8N (*red*), and 16N (*black*). Cysts that did not stain at all are marked in *yellow*. AZT, azidothymidine; DMSO, dimethyl sulfoxide; FDA, fluorescein diacetate; PI, propidium iodide.

**Figure 8:**
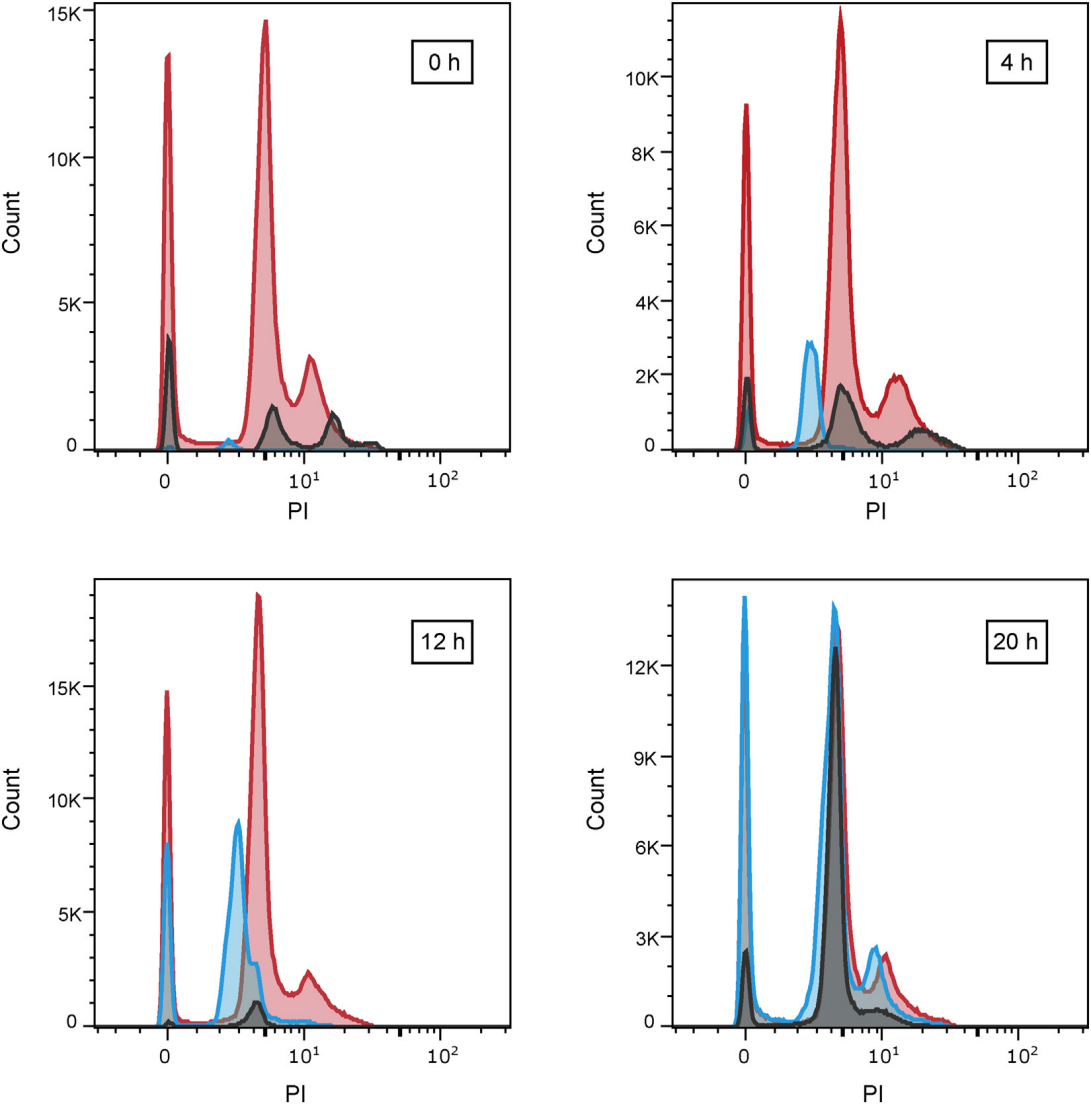
**PI signals at different time points in the cysts after control, AZT, and metronidazole treatments.** Cysts were recovered from cells treated at different time points during encystation with AZT, metronidazole, or pure encystation medium with the PI-staining indicated. The resulting fluorescence profiles were overlaid upon each other and show a clear reduction of cell numbers for both drugs at early time points and a pronounced shift of PI signals from AZT at all but the last time point. The control is in *red*, AZT treatment is in *blue*, and metronidazole treatment is in *gray*. AZT, azidothymidine; PI, propidium iodide.

The Uppsala encystation protocol (25) was used with *G. intestinalis* cells to investigate the effect of AZT on cyst formation. As a final step, cysts were stored in ddH_2_O for 3 days to lyse cells that had not initiated the encystation process and to open the cell membranes of immature cysts to make them permeable to propidium iodide (PI), which allowed measurements of their DNA contents. A second dye, fluorescein diacetate (FDA), detects living cells and stains cysts that have completed the encystation process and that are impermeable to PI (FDA^+^, PI^−^). In this way, intact cysts become clearly separated from immature cysts (FDA^−^, PI^+^) that are further categorized depending on how many times the genome has been amplified (Fig. S6). In our AZT experiments, the drug was added at certain time points during the encystation process, and the results were compared with similar experiments performed with metronidazole. We also included two controls, one with pure encystation medium and one that contained a similar final concentration of the solvent (dimethyl sulfoxide [DMSO]) as in the drug-treated cells. There was little variation in the number of cysts or the staining pattern between the two controls apart from a clear dip around 8 h (Fig. 7). At this time point, cells undergo membrane reorganization and are particularly sensitive to any disturbance (25). Only at the time points of 8 h and 12 h could cells that were not labeled with either dye be detected at significant levels. These cells either did not contain DNA or had intact membranes but lacked FDA-activating esterases. When comparing AZT-treated cells with the two controls, a strong effect was observed if AZT was added early during encystation (0–8 h), with almost complete removal of cysts when treating immediately upon induction of encystation (0 h). This is in good agreement with the experiments in Figure 6 showing that AZT blocked DNA synthesis. Another observation was the increased percentage of cysts with lower genome duplication levels (notably 4N) when comparing controls to AZT-treated cells at all time points except 20 h when most DNA synthesis had been completed prior to the addition of the drug (Fig. S7). Further analysis showed that the increased percentage of 4N cells was because the 8N peak shifted to the left and therefore became partially categorized as 4N (Figs. 8 and S8). These results suggest that the genome amplification to 8N was incomplete, which is in line with the mechanism of AZT as a chain terminator of DNA synthesis creating sequence gaps and thereby lowering the DNA content per cell. This explains the decreased effect of AZT at later time points when DNA synthesis is almost completed.

Experiments with metronidazole showed a different kind of pattern, and cell numbers were reduced substantially at all time points (Fig. 7). However, the effect was less pronounced compared to AZT at 0 h and there was no peak shift to the left (Figs. 8 and S8). This is in line with the respective mode of drug action in which metronidazole kills anaerobic cells directly *via* radical induced damage, whereas AZT blocks DNA replication and thereby prevents proliferation and proper encystation.

### Antigiardial activity of AZT in a rodent model

**Figure 9:**
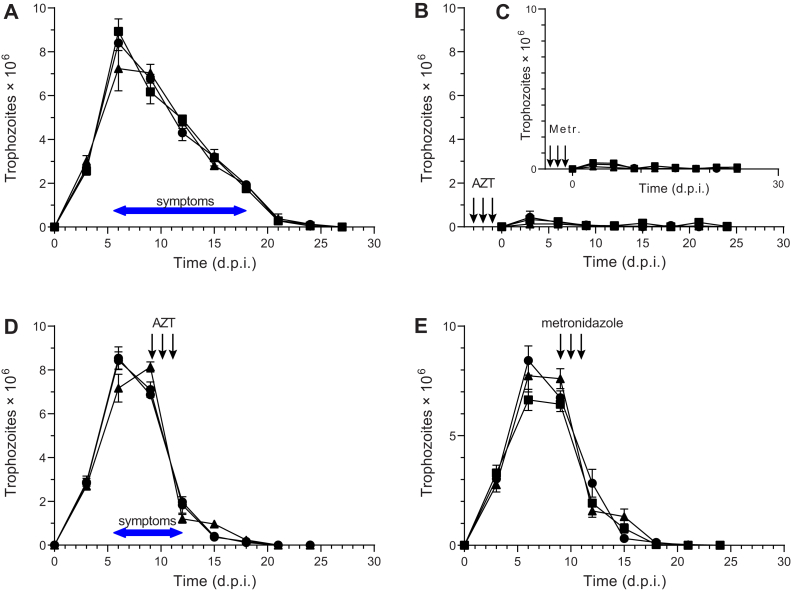
**AZT treatment of *G. intestinalis*–infected gerbils.***A*, the effect of PBS (control) on the number of trophozoites in the intestines of gerbils infected with the different *G. intestinalis* strains GS/M-H7 (●), WB-417 (▲), or WB-1267 (■). *B* and *C*, the number of intestinal trophozoites in gerbils treated with AZT or metronidazole for 3 days prior to the infection. *D*, the number of intestinal trophozoites in gerbils treated with AZT for 3 days starting at day 10. *E*, similar to (*D*) but with metronidazole treatment. The period of symptoms is also shown in (*A*) and (*D*) with a more detailed summary of symptoms in Table S1. AZT, azidothymidine.

The gerbil model of acute giardiasis was used to assess the activity of AZT on three different *G. intestinalis* cell lines, including two assemblage A clones (WB-1267 and WB-417) expressing VSP1267 and VSP414, respectively, and one assemblage B clone (GS/M-H7) expressing VSPH7. Six-week-old male and female gerbils were orogastrically inoculated with 5 × 10^5^ trophozoites of each of the three clones, and the magnitude of infection was verified either by counting the trophozoites collected from the upper portions of the gerbils’ small intestine or by quantification of cysts in the stools over a 30 day period, as previously reported (26). Oral administration of 40 mg/kg AZT (treated), 25 mg/kg of metronidazole (treated control), or PBS alone (untreated controls) for 3 days as a pretreatment before the infection showed a marked effect on the infectivity of each of the clones compared to the untreated controls (Fig. 9, *A*–*C*). The number of trophozoites within the intestine and the number of cysts in the stool were negligible in the animals treated with AZT or metronidazole (Figs. 9, *B* and *C* and S9, *B* and *C*). In experiments where the 3 day AZT treatment was instead started at the peak of infection (day 10 postinfection), this resulted in a rapid decrease in parasite burden and a drastically reduced period with symptoms (Fig. 9*D*) as compared to the control gerbils (Fig. 9*A*). The numbers of cysts were also drastically reduced upon treatment, albeit with a delay of a few days (Fig. S9*D*). The infection gradually leveled off over time also in the control animals without treatment, which is typical for self-healing infections, but in the case of the AZT-treated gerbils, the effect was much faster and the symptomatic period much shorter. There was no obvious difference between the effects of AZT and metronidazole, and both drugs seemed equally efficient in reducing parasitemia and cyst formation (Figs. 9, *B*–*E* and S9, *B*–*E*).

## Discussion

**Figure 10:**
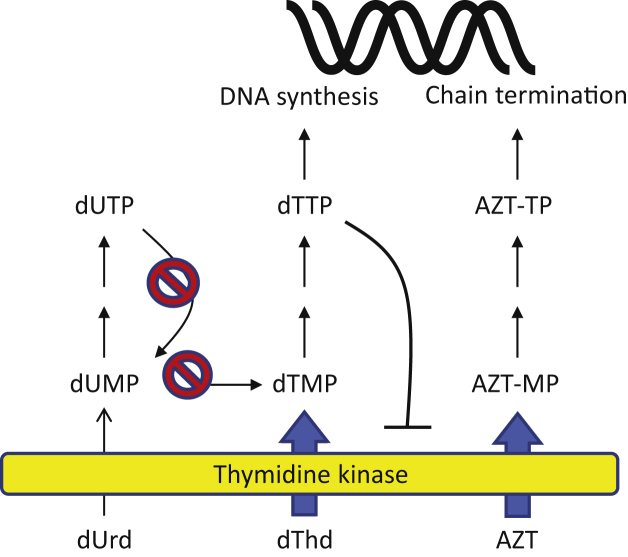
**Role of *G. intestinalis* thymidine kinase in parasite DNA synthesis and drug discovery.** The widths of the *arrows* indicate which substrates are preferred by the thymidine kinase, with thymidine and AZT being much more efficient than deoxyuridine. Thymidine phosphorylation leads to the formation of dTTP, which is used as a building block for DNA replication, whereas AZT is a drug that is metabolized in a similar manner as thymidine but acts as a chain terminator in DNA synthesis. The figure also shows two enzymes missing in *G. intestinalis*, dUTPase (dUTP → dUMP) and thymidylate synthase (dUMP → dTMP). AZT, azidothymidine.

This work provides insights into the biological role of the *G. intestinalis* thymidine kinase and how its substrate selectivity can be exploited for drug discovery (Fig. 10). One of the most striking findings when studying the isolated enzyme was the large difference in affinity for deoxyuridine in comparison to thymidine. The *G. intestinalis* enzyme had a catalytic efficiency that was ∼100 times higher with thymidine compared to deoxyuridine. For comparison, there is only a 14-fold difference between the two substrates with the mammalian thymidine kinase (TK1) (23). The main difference between the two enzymes is the affinity for thymidine, as indicated by their *K*_*m*_ values of 0.07 μM and 0.5 μM, respectively. The stronger discrimination between thymidine and deoxyuridine in the *G.intestinalis* enzyme is just a consequence of its higher thymidine affinity. This could be an adaption to be able to grow under conditions with low thymidine concentrations, but also to survive in the absence of dUTPase, an enzyme that the parasite lacks. The incorporation of dUTP into DNA is a major cause of abasic sites created by base excision repair (27), and dUTPase is therefore needed in most organisms. *G. intestinalis* is a special case because it lacks ribonucleotide reductase making phosphorylation of deoxyuridine the only source of dUTP. It is also important to remember that deoxyuridine monophosphate (dUMP) formed in *G. intestinalis* is a dead-end product. In most other organisms, dUMP is the substrate of thymidylate synthase and can thereby be channeled into dTTP production. *G. intestinalis* lacks this enzyme and cannot use dUMP. Hence, it is logical that the enzyme has developed a much stronger selectivity of thymidine over deoxyuridine.

The *G. intestinalis* thymidine kinase and other members from the TK1 family are regulated by dTTP, which binds to the active site and competes with thymidine. This kind of regulation seems counterintuitive from a single cell perspective because it decreases inhibition when there is plenty of thymidine and would thus not seem optimal for controlling the intracellular concentration of dTTP at a stable level. We therefore think the main benefit of this regulation is to save thymidine for the neighboring cells when there are limited amounts of thymidine in the surroundings. For *G. intestinalis*, which is a single-cell organism, it could be an advantage to spread the thymidine equally between cells of the same clone.

A major purpose of the current study was to find an alternative class of medicines for *G. intestinalis* infections. Our search was prompted by the realization that there are only two classes of first-line drugs targeting this parasite: 5-nitroimidazoles (including metronidazole) and the closely related benzimidazole albendazole (28). The close relation of the two main drug classes against giardiasis raises the concern of multidrug resistance, especially based on the recent reports of up to 40% treatment failures by metronidazole (10). The only other approved drugs, paromomycin and quinacrine, have been second lined due to their side effects, costs, and administration difficulties making them unusable in resource-poor settings where giardiasis is most severe (28). An ideal drug against *G. intestinalis* should fulfill the following criteria:

(1) Strong effect against the parasite and prevention of viable cyst formation and thereby transmission.
(2) Easy form of administration to facilitate intake in low-resource settings.
(3) Good safety profile, which means few and well-characterized side effects.
(4) Low costs.

The last two points are hard to combine when developing an antiparasitic drug from scratch (29), and we were therefore focusing on drugs that have been explored for other diseases (30). When looking for new treatment options for a given disease, it is important to keep in mind in which setting the disease is prevalent. Giardiasis is a disease infecting disproportionally more people in the developing world (1), so treatment options have to be inexpensive if they are to be widely used. Clinical trials represent a major cost associated with drug development (31), and thus, it is advisable to establish first whether a drug that has been developed and approved by the Food and Drug Administration for another purpose can be used for the condition instead (30). In lower-resource settings non-orally administered drugs can be harder to promote, thus putting extra emphasis on selecting a drug that can be taken orally. Keeping the low-resource environment in mind, the drug should not require cold storage if possible.

We set out to identify a potential drug that fulfills these requirements and found that the well-established anti-HIV drug zidovudine (AZT) was a good substrate for the *G. intestinalis* thymidine kinase and efficiently inhibited parasite DNA synthesis and proliferation. AZT is a thymidine analog with an azido group replacing the 3′ OH group, and it remains a choice in HIV treatment today, usually as part of a combination treatment (32). *Giardia* infections were frequently seen during the early stages of the HIV pandemic, but after introduction of combination therapies containing AZT the levels decreased (33). AZT is also widely used for HIV treatment during pregnancy and during birth to prevent mother-to-child transmission (34). The drug is administered orally, and peak plasma levels as high as 2 to 18 μM are generally well tolerated (35, 36). Short-term dosing studies showed that serum concentrations up to 133 μM were safely reached for 48 h, indicating that the therapeutic window is large (37). Heavy side effects seem limited to long-term use, combination use, and use of AZT in patients with advanced HIV-1. In those settings, effects like hematologic toxicity, myopathy, and hypersensitivity can be observed. However, over short-term use only mild anemia, headaches, nausea, and other sorts of intestinal discomfort have been reported. Given that our studies in gerbils showed that the infection resolved with a 3-day treatment schedule, long-term side effects will most likely not be of concern in the treatment of giardiasis with AZT (38). Median costs during treatment for HIV are ∼80 USD per month with the drug being taken twice daily, which corresponds to a price of ∼1.3 USD per 300 mg tablet (https://apps.who.int/iris/bitstream/handle/10665/104451/9789241506755_eng.pdf?sequence=1&isAllowed=y).

We conclude that AZT is a potential alternative to metronidazole because it fulfills all the criteria that we established initially for an antigiardial drug to be successful. AZT has a different mode of action compared to the 5-nitroimidazoles, and it efficiently kills the trophozoites of *G. intestinalis* both *in vitro* and *in vivo* at a concentration comparable to metronidazole. AZT also reduces the number of cysts formed *in vitro* and *in vivo*. Furthermore, experiments on cell lines resistant to metronidazole, tinidazole, ornidazole and nitazoxanide showed that there is no indication for crossresistance with metronidazole and related compounds. AZT has the Food and Drug Administration approval and is priced rather cheaply due to its widespread use as an anti-HIV medicine. The common use also gives us a rich body of information on side effects, which are expected to be low with the short treatment period proposed here. Another positive aspect of repurposing an existing drug is the existing production and distribution lines reaching many low-resource settings. Finally, the drug can be taken orally and stored at room temperature (RT), thus enabling its administration in most places without additional equipment or education. In conclusion, AZT appears to be a promising candidate to treat both 5-nitroimidazole refractory and nonrefractory giardiasis.

## Experimental procedures

### Phylogenetic analyses

*G. intestinalis* assemblage A isolate WB thymidine kinase was used as a BLASTp query against the NCBI nr database (June 2020), keeping 500 hits with *e*-values <0.001. The resulting sequences were filtered using CD-HIT (39) by keeping sequences with <90% sequence identity to another sequence in the dataset. The resulting dataset was aligned using MAFFT v6.603b (40) with the default setting and trimmed using BMGE (41) v.1.12 (BLOSUM30 with a block size of 2). A preliminary tree was computed using FastTree (42) v2.1.8 SSE3 OpenMP with the default setting, and sequences with a phylogenetic distance <0.3 were removed in an iterative process until the final dataset was generated.

The final dataset was aligned using MAFFT and trimmed using BMGE as described previously. A maximum likelihood tree was computed using IQtree (43) v.1.5.3 under the LGX substitution model. Branch supports were assessed using ultrafast bootstrap approximation (UFboot) (44) with 1000 bootstrap replicates and SH-like approximate likelihood ratio test (SH-aLRT) (45), for which 1000 replicates were used.

### Cloning of the *G. intestinalis* thymidine kinase gene for expression in *E. coli*

The identified *G. intestinalis* thymidine kinase gene was from assemblage A isolate WB (GL50803_8364) in the Giardia genomic resource (https://giardiadb.org/giardiadb/). The gene was amplified from genomic DNA using the following primers: 5′- GCC **TCA TGA** ACT CCC TTA CGC TCA TAC TGG -3′and 5′-CAT GCT **GGT ACC** TCA CAG GTC TTC GTT GAT CCC TAG CA-3′. To enable cloning, the primers contained the restriction sites BspH1 and Acc65I, respectively (bold-faced). The amplified thymidine kinase sequence was cloned into the expression vector pET-Z. The resulting vector, pET-ZTK, contained the 10 kDa fusion partner Z (Z domain of staphylococcal protein A), a His tag, and a tobacco etch virus (TEV) site prior to the inserted gene. Positive clones were confirmed by DNA sequencing from both directions of the insert.

### Protein expression in *E. coli* and purification

The pET-ZTK vector was introduced into *E. coli* BL21(DE3)plys, and the bacteria were grown in 1 l LB medium supplemented with 50 μg/ml kanamycin and 34 μg/ml chloramphenicol at 37 °C. Expression of the *G. intestinalis* thymidine kinase was induced at *A*_600_ ≃0.6 with 0.5 mM IPTG overnight at 18 °C. The cells were harvested by centrifugation at 4000*g* for 15 min. The pellet was washed with 20 mM Tris–HCl pH 7.5 and centrifuged at 4000*g* for 10 min. The supernatant was discarded, and the pellet was resuspended in lysis buffer (20 mM Tris–HCl pH 7.5, 0.4 M NaCl, and 0.1 mM PMSF). The volume used was then 10 ml/g of pellet. The cell suspension was frozen and thawed three times to lyse the cells followed by centrifugation at 40,000 rpm for 45 min (Beckman L-90 ultracentrifuge Ti70 rotor). The supernatant was loaded onto a 2 ml Millipore Ni-NTA His·Bind resin column (Merck). The purification steps were as follows. The column was first washed with 20 mM Tris–HCL pH 7.5, 0.4 M NaCl, and 20 mM imidazole. The second wash solution was similar but with 50 mM imidazole. The Z-tagged thymidine kinase was finally eluted with 20 mM Tris–HCl, 150 mM NaCl, and 250 mM imidazole. This protein contained the His-tagged Z fusion partner linked with a TEV protease site to the thymidine kinase. In order to remove the fusion partner, the protein was first buffer-exchanged into a solution containing 20 mM Tris–HCL pH 7.5 and 150 mM NaCl using Sephadex G-25 chromatography to remove the imidazole. Subsequently, TEV protease was added to the Z-tagged thymidine kinase at a ratio of 1:10 for 3 h at RT. The mixture was finally loaded onto a 0.4 ml Ni-NTA His·Bind resin, and the cleaved thymidine kinase protein was eluted with a solution containing 20 mM Tris–HCl pH 7.5, 150 mM NaCl, and 20 mM imidazole. The protein solution was mixed 1:1 with a 50% (v/v) glycerol solution and stored at −80 °C and aliquoted into 50 to 100 μl fractions because of its tendency to aggregate after several freeze-thaw cycles.

### Thymidine kinase assay

*G. intestinalis* thymidine kinase was incubated in 50 μl of buffer containing [5′-^3^H]-labeled thymidine (Moravek Biochemicals), 2 mM ATP, 5 mM MgCl_2_, 0.5 mM DTT, 100 mM potassium acetate, and 50 mM Tris–HCl, pH 7.5, at 37 °C for 30 min. In one sample for each concentration of thymidine measured, the enzyme was omitted to determine background counts. In experiments where we replaced ATP with different concentrations of phosphate donors (CTP, UTP, GTP, and ATP), the MgCl_2_ concentration was increased to 10 mM. After the enzyme reaction was completed, the samples were incubated at 100 °C for 2 min to stop the reaction. The remaining procedure was similar to what was described previously (46). The reaction mix was spotted onto two Whatman DE81 filters from GE Healthcare (20 μl was spotted on each filter). Because this product was discontinued, we could only use it initially and in later assays we replaced it with Amersham Hybond-XL (GE Healthcare), which we cut into 15 × 15 mm squares. The highest usable concentration of phosphate donor to avoid saturation of the filter was 5 mM, but it was possible to also use up to 10 mM if the sample was diluted twofold prior to spotting. After applying the samples, the filters were dried for at least half an hour in darkness to minimize light-induced background counts. Subsequently, the filters were put into separate scintillation vials, washed three times with 5 ml of 1 mM ammonium formate with agitation for 5 min each time (decanting the liquid after each wash). The final wash was with 5 ml EtOH, and after decanting the liquid, we added 1 ml of a solution containing 0.1 M HCl and 0.1 M KCl and vortexed the tubes for 20 s. Finally, 5 ml scintillation liquid was added and the samples were mixed by shaking the tubes before radioactivity counting. Similar results were obtained on both types of filters.

An alternative way of measuring the thymidine kinase activity was used in the five-substrate assay, where thymidine, deoxyuridine, deoxycytidine, deoxyadenosine, and deoxyguanosine were present simultaneously. In this case, the substrates were nonradioactive and the amount of product was measured by HPLC. The HPLC protocol is a variant of a previously described protocol for nucleoside triphosphates (47) but with a lower concentration of phosphate in the mobile phase in order to optimize it for the study of monophosphates instead of triphosphates and with methanol instead of acetonitrile in order to create larger spacing between the peaks. The assay itself was performed as described previously and included 200 μM of each of the five substrates and 100 ng *G intestinalis* thymidine kinase. After the inactivation step (boiling for 2 min), 20 μl of the reaction mixture was diluted nine times and mixed with 10× loading buffer (described later). The mixture was loaded onto a 150 mm × 4.6 mm SunShell C18-WP column from ChromaNik Technologies Inc. The mobile phase was a ternary mixture of three solutions; A, B, and C. Solution A contained 23 g/l potassium phosphate in 7% (v/v) methanol and was adjusted to pH 5.6 with 1.27 ml 4 M KOH per liter, solution B contained only 7% methanol, and solution C contained 3.52 g/l tetrabutylammonium bromide in 7% methanol. The 10× loading buffer was prepared by mixing 500 μl 4× solution C (1.41 g tetrabutylammonium bromide in 100 ml water) with 500 μl solution A. The column was run at ambient temperature and equilibrated with 11% solution A, 69% solution B, and 20% solution C. The flow rate was 1 ml/min, the loop size was 100 μl, and the UV detector was set at 270 nm. The deoxyribonucleoside substrates and deoxynucleoside monophosphate (dNMP) products were eluted isocratically under these conditions. After the last peak of interest, deoxyadenosine monophosphate (dAMP) had eluted at ∼20 min; the composition was changed to a mixture of 70% A, 10% B, and 20% C to elute ATP before returning to initial conditions. A 10 min equilibration step was included between the runs. The dNMPs were quantified by comparing the peak heights to a standard with known concentrations and multiplied by 10 to compensate for the previous dilution step.

### Mass photometry analysis

Analyses were performed on a Refeyn 2^MP^ mass photometer (Refeyn Ltd) calibrated with NativeMark Unstained Protein Standard (Thermo Fisher Scientific). The thymidine kinase was mixed with PBS giving a final protein concentration of 50 nM prior to analysis.

### Cell maintenance of parasites used for *in vitro* assays

The following *G. intestinalis* isolates, which were induced for *in vitro* resistance toward metronidazole according to Tejman-Yarden *et al.* (21), were used in the *in vitro* assays: WB (ATCC 50803) clone C6/A11 (WB) and the recently generated strains WB-C6/A11-M1 (M1), WB-C6/A11-M2 (M2), and WB-C6/A11-M1NR (M1NR). Cells were cultured in TYI-S-33 medium as described elsewhere (48). The metronidazole-resistant strains were continuously maintained in medium supplemented with metronidazole, which was removed in the last passage (24 h) before the experiments.

### Drug susceptibility assays

To establish drug efficiency, EC_50_ values were determined. Briefly, cells were counted and diluted to 1 × 10^4^ cells/ml for metronidazole-sensitive cells and 2 × 10^4^ cells/ml for the resistant lines and seeded in 40 μl TYI-S-33 per well in 96-well plates. These values were adjusted to 5 × 10^3^ cells/ml for sensitive cells and 1 × 10^4^ cells/ml for resistant lines when incubating for 72 h. The 96-well plates were inoculated anaerobically at 37 °C for 2 h to allow the cells to attach. Drugs were diluted in growth medium from 200 mM stocks in DMSO to their final concentrations. After 2 h, 160 μl TYI-S33 containing the drug at concentrations of 1 to 50 μM for AZT and 1 to 200 μM for metronidazole were added. For every drug concentration a control well with corresponding DMSO dilutions was run to correct for solvent effects. Plates were incubated for 48 h or 72 h anaerobically at 37 °C before adding 50 μl CellTiter-Glo reagent (Promega). The plates were mixed by shaking for 10 min, the mixture was allowed to settle for 10 min, and the fluorescence was read out in an Infinite M200 Pro (Tecan Group, Ltd). EC_50_ values were determined by fitting the data to Log inhibitor *versus* Response curves (four parameters, variable slope) using GraphPad Prism 9.3.1 (GraphPad Software Inc). Standard errors were then determined as the variation between the individual EC_50_ values from three or four independent experiments.

### Inhibition of DNA synthesis

*G. intestinalis* trophozoites at 70% confluence were either incubated with 2 μCi [5-^3^H(N)]-deoxycytidine (10.5 nM) or a combination of 4 μCi [2,8-^3^H]-deoxyadenosine (13 nM) (Moravek Biochemicals) and 2 μM deoxycoformycin in the presence of various concentrations of AZT in a 10 cm culture dish containing 10 ml cell culture medium for 1 h. The culture medium was aspirated after 1 h anaerobic incubation at 37 °C, and the cells were washed with PBS before the addition of 500 μl ice cold 10% (w/v) trichloroacetic acid (TCA) containing 15 mM MgCl_2_ (referred to as TCA-MgCl_2_ solution). The cells were disintegrated with a cell scraper, and the DNA was recovered essentially as described previously (49). The solution was centrifuged for 1 min at 21,000*g*, and the supernatant was discarded. The pellet was washed with 200 μl TCA-MgCl_2_ solution, and after aspiration of the washing solution, the pellet was resuspended in 500 μl of 0.6 M NaOH and incubated overnight at 37 °C. Subsequently, 400 μl TCA was added and the resulting solution was filtered through a 24 mm Whatman GF/C 24 filter (GE healthcare). The filter was put in a vial, 5 ml scintillation liquid was added, and the mixture was vortexed for 30 s before counting in a scintillation counter to measure the radioactivity in the DNA.

### Inhibition of encystation

Encystation inhibition assays were performed in triplicate in 3 ml Nunc cell culture tubes #156758 (Thermo Fisher Scientific). Cells of the WB line were counted in a hemacytometer and diluted to ∼2.5 × 10^5^ cells. They were then incubated in TYI-S-33 at 37 °C for 3 h to allow them to attach again before the medium was changed to encystation medium according to the Uppsala encystation protocol (25). At five time points post encystation induction (0 h, 4 h, 8 h, 12 h, or 20 h), drugs were added in 10 μl DMSO at a final concentration of 53 μM (metronidazole) and 60 μM (AZT), and the tubes were inverted several times to ensure distribution of the drug. Control tubes, to which nothing was added, were opened, closed, and inverted for the same amount of time as the other tubes. For the DMSO control, 10 μl of sterile DMSO was added. The concentrations of the selected drugs were chosen to be 10× the EC_50_ at 48 h based on the results of Hausen *et al.* (50) for metronidazole, where they showed a clear effect when added early but not late during encystation at that concentration, as well as the reports of peak serum concentrations for common dosage regimens between 30 and 70 μM (51, 52).

After 28 h incubation at 37 °C, the cells were harvested by centrifuging at 800*g* for 8 min at 4 °C, washed twice with sterile H_2_O, and incubated in sterile H_2_O at 4 °C for 3 to 5 days. Cysts were live-stained/dead-stained with FDA and PI similarly to what was described for *G. muris* cysts (53). FDA was dissolved into a stock solution of 25 mM in acetone and kept in the dark. Immediately before incubation, it was diluted to a working solution by adding 80 μl to 10 ml PBS pH 6. The cysts were spun down, resuspended in 60 μl PBS, and stained by the addition of 40 μl PI Ready Flow Reagent (Thermo Fisher Scientific) and 100 μl FDA working solution. Cells were incubated for 30 min at RT in the dark before counting them in a MACSQuant VYB cell counter (Miltenyi Biotec Inc) using the blue laser (488 nm) and B1 filter (525 nm/50 nm) for FDA and the yellow laser (561 nm) and Y2 filter (615 nm/20 nm) for PI. For each drug, three biological replicates were analyzed, and half of the total 200 μl volume of each sample was counted. Typical staining and graphs are shown in Fig. S6.

### Parasites used in animal experiments

*G. intestinalis* WB (ATCC 50803) and GS/M (ATCC 50581) clones derived from the WB and GS strains, respectively, were cultured at 37 °C in TYI-S-33 medium in 14 ml borosilicate screw-cap glass tubes (54). *Giardia* clones expressing different surface antigens were obtained by limiting dilution using specific anti-VSP mAbs (54). Reactive clones were expanded in culture medium overnight and controlled for homogeneity before use. WB clones 1267 (mAb 5C1), 417 (mAb 7C2), and GS/M-H7 (mAb G10/4) were employed in control experiments and treatments.

### Animal experiments—ethics and general maintenance

The animal studies were approved by the ethics committee in Cordoba, Argentina. All procedures performed on animals were conducted following protocols specifically approved by the Institutional Committee for Care and Use of Experimental Animals (protocol 2010-36-15p). These protocols adhered to the US PHS guidelines for animal research, and no animals were harmed during the collection of blood, fecal samples, or intestinal contents.

Specific pathogen-free, 6-week-old outbred gerbils (*Meriones unguiculatus*) of both sexes were used (Animal Facility CIDIE CONICET/UCC). They were housed in air-conditioned (18–22 °C, 40–50% humidity) racks with a 12 h light–dark cycle. Gerbils were fed *ad libitum* with autoclaved food and sterile water supplemented with a mixture of filter-sterilized vitamin solution. Before infection, gerbils were tested for the presence of serum antibodies against *Giardia* antigens by ELISA and for *Giardia* cysts in stool samples by light and immunofluorescence microscopy using cyst-specific mAb 7D6, as previously reported (55). In infection and treatment experiments where the number of trophozoites was counted, each experimental series required ten gerbils with one animal sacrificed for each data point (a total of ten time points per series), whereas in the experiments where cysts were counted, only one gerbil per condition and *G. intestinalis* strain was required. There were totally five different conditions (PBS control and two conditions with each drug), three *G. intestinalis* strains for each condition, and three independent series. The experiments where cysts were counted had a similar setup but required much fewer animals because each one could be used for all ten time points. In a separate study to monitor symptoms, three conditions (treatment with PBS and two AZT treatment regimens) were tested on gerbils infected with each of the three *G. intestinalis* strains and on uninfected animals. Also in this case, the data were based on three independent experiments.

### Animal experiments—infection and treatment

#### Infections

Gerbil infections were induced by orogastric inoculation of 2 × 10^5^ trophozoites resuspended in 0.5 ml PBS (55). Control gerbils received 0.5 ml PBS by the same route. Feces were collected every day from week 0 to 4. Cysts were identified visually by light microscopy and by immunofluorescence assays with mAb 7D6. *Giardia* cysts excreted by gerbils were quantified by collecting stool pellets from individually housed gerbils over a 24 h period. The stool samples were weighed, resuspended in 2 ml of PBS, and filtered. The filtrate was centrifuged at 250*g* for 10 min. After three washes, the pellet was suspended in 2 ml PBS and cysts were stained and counted in a hemacytometer (55). The following criteria were used for considering animals to be uninfected: no cysts were found in the feces, stool samples were unable to infect uninfected gerbils, and no trophozoites were detected after 6 days in culture with anticyst mAbs (55).

#### Trophozoite recovery

At the indicated times postinfection, the gerbils were euthanized in a CO_2_ chamber and the first portion of the small intestine (10 cm) was removed and placed in TSY-S-33 medium on ice. The intestinal pieces were minced and placed on ice for 30 min and then the parasites were counted with a hemacytometer. Trophozoites were identified visually *via* immunofluorescence assays using a trophozoite-specific antigen (mAb 9C9 anti-BiP (26, 56)). Excreted *Giardia* cysts were counted by collecting stool samples from individually housed animals over a 24 h period, as reported previously (26).

#### Treatments

Gerbils were fasted for 6 h and weighed before orally administering by gavage either PBS (controls), AZT at 40 mg/kg body weight for each dose, or metronidazole at 25 mg/kg body/weight for each dose at the indicated time points. Two experimental approaches were used. First, either AZT, metronidazole, or PBS was administered daily for 3 days prior to infections. Second, the drug or the vehicle was administered for 3 days starting at day 10 postinfection. The effects of the treatments were determined by counting the cysts in the stool samples of individual gerbils and by euthanizing one animal every 3 days to collect the trophozoites present within the small intestine, as described previously. In independent experiments, the clinical signs of the infections under the different treatments were evaluated daily.

## Data availability

All data are contained in the main article and supporting material.

## Supporting information

This article contains supporting information.

## Conflict of interest

The authors declare that they have no conflicts of interest with the contents of this article.
