# Supplementary material for: Giardia intestinalis thymidine kinase is a high-affinity enzyme crucial for DNA synthesis and an exploitable target for drug discovery
Source: J Biol Chem. 2022 May 11;298(6):102028. doi: 10.1016/j.jbc.2022.102028 (PMC9190010; doi:10.1016/j.jbc.2022.102028)
Supplement: Supplemental Figures S1–S9 and Table S1 [file mmc1.pdf]

Supporting information

Table of content. Note that the figure titles are abbreviated.

Figure S1. Amino acid sequence alignment of thymidine kinases S-2

Figure S2. Phylogenetic analysis of *G. intestinalis* thymidine kinase S-3

Figure S3. Effect of dithiothreitol on *G. intestinalis* thymidine kinase S-4

Figure S4. Linearity of the *G. intestinalis* thymidine kinase assay S-4

Figure S5. Inhibition of *G. intestinalis* proliferation S-5

Figure S6. Illustration of the procedure for categorizing cysts S-6

Figure S7. Effects of AZT and metronidazole on cyst development S-7

Figure S8. Effect of AZT and metronidazole on the DNA content of cysts S-8

Figure S9. Number of cysts generated in treated *G. intestinalis*-infected gerbils S-9

Table S1. Signs of giardiasis in treated *G. intestinalis*-infected gerbils S-10

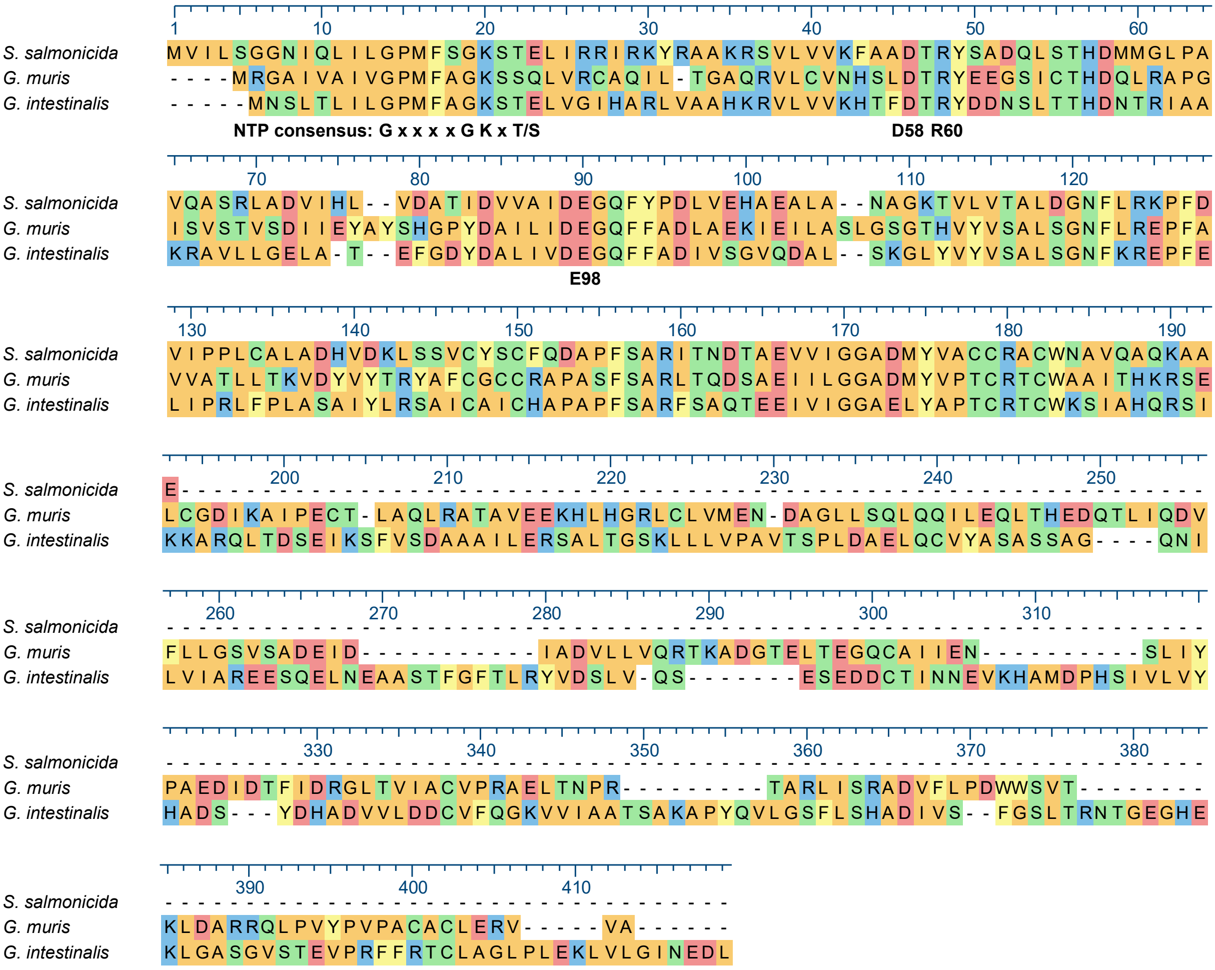

Figure S1. Amino acid sequence alignment of thymidine kinases from three diplomonads. The alignment was generated with Clustal Omega, and the color code indicates positive charge (blue), negative charge (red), non-polar residues (orange), polar residues (green), and aromatic residues (yellow). Highlighted sequences are the NTP consensus sequence needed for ATP binding, D58 needed for interaction with the 3´-hydroxyl group of the deoxyribose, R60 needed for the formation of the transition state, and E98 needed for abstraction of the hydrogen from the 5´-hydroxyl group to initiate the enzymatic reaction.

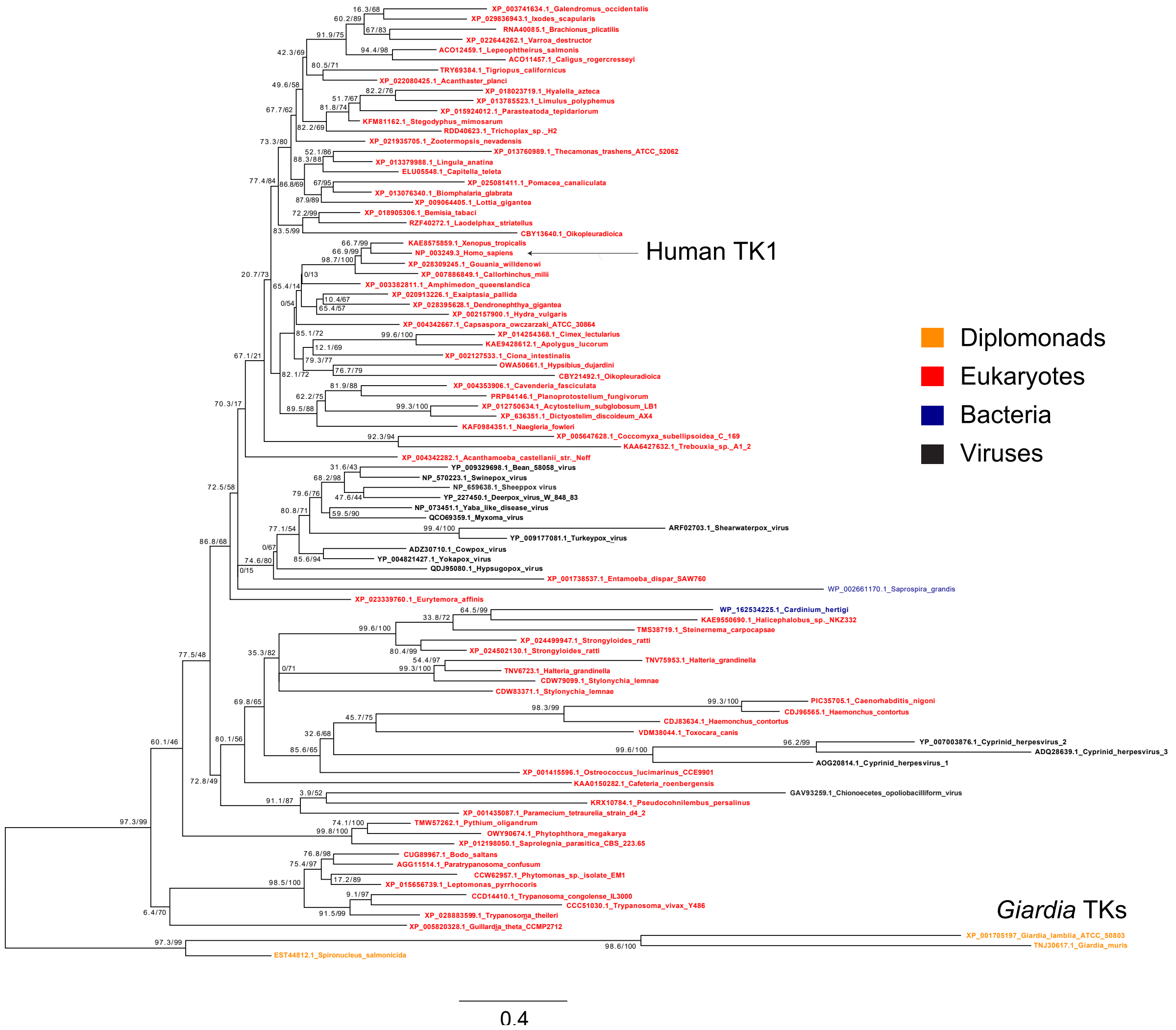

Figure S2. Phylogenetic analysis of *G. intestinalis* thymidine kinase. The phylogenetic tree was created using *G. intestinalis* thymidine kinase in a BLASTp search against the nr database from NCBI (*e*-value <0.001 and maximum number of sequences 500). Close relatives (>90% of sequence identity or a phylogenetic distance <0.3) to the shown species were discarded prior to the tree building. The resulting thymidine kinase tree gives a representable coverage of TK1 members from eukaryotes and associated viruses, whereas most bacterial/archaeal thymidine kinases fall outside the selection criteria. The branch support values were assessed using ultrafast bootstrap approximation (UFboot) with 1,000 bootstrap replicates (42) and a SH-like approximate likelihood ratio test (SH-aLRT) (43), for which 1,000 replicates were used.

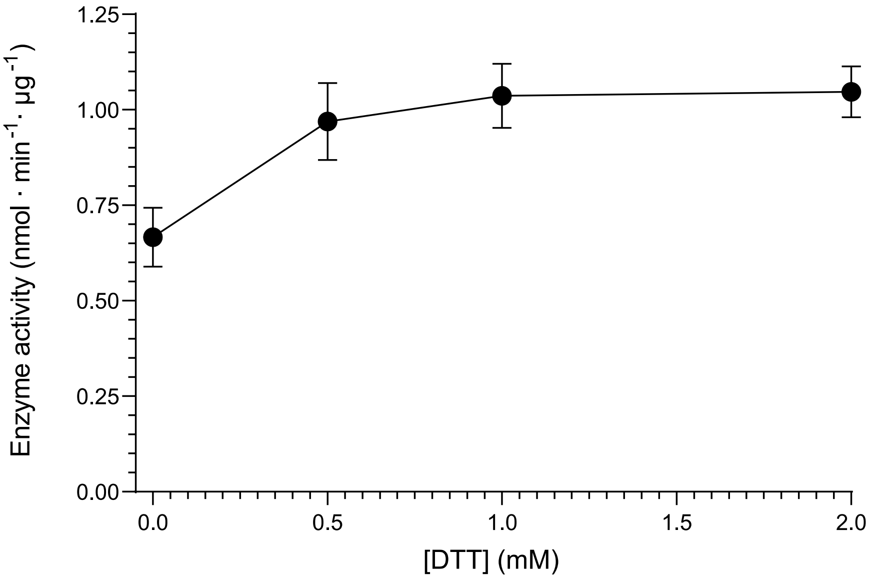

Figure S3. The effect of dithiothreitol on *G. intestinalis* thymidine kinase activity. The enzyme assay was performed with 200 µM thymidine and standard conditions regarding all other aspects. The data are from three independent experiments with standard errors shown.

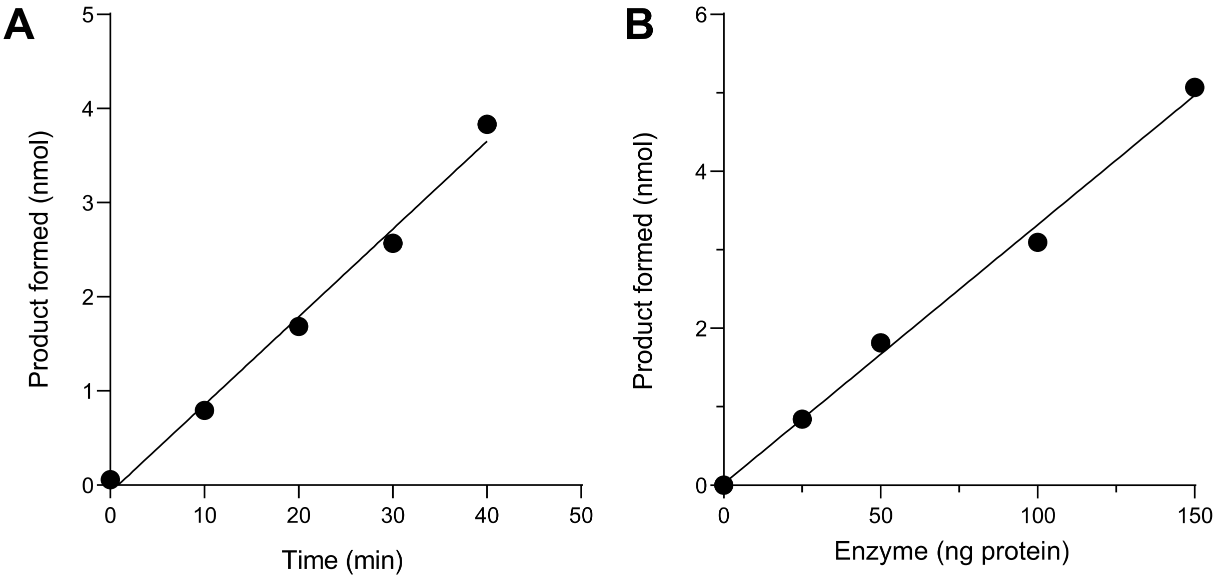

Figure S4. Linearity of the *G. intestinalis* thymidine kinase assay. (A) The enzyme assay was linear with respect to temperature in the 40-minute interval tested. (B) The assay was linear with respect to enzyme amount up to 150 ng. The assays were performed with 200 µM thymidine, 100 ng enzyme in A (variable in B), and 30 min in B (variable time in A) using standard conditions regarding all other aspects. Curves are fitted by linear regression giving R^2 values of 0.99 in both A and B.

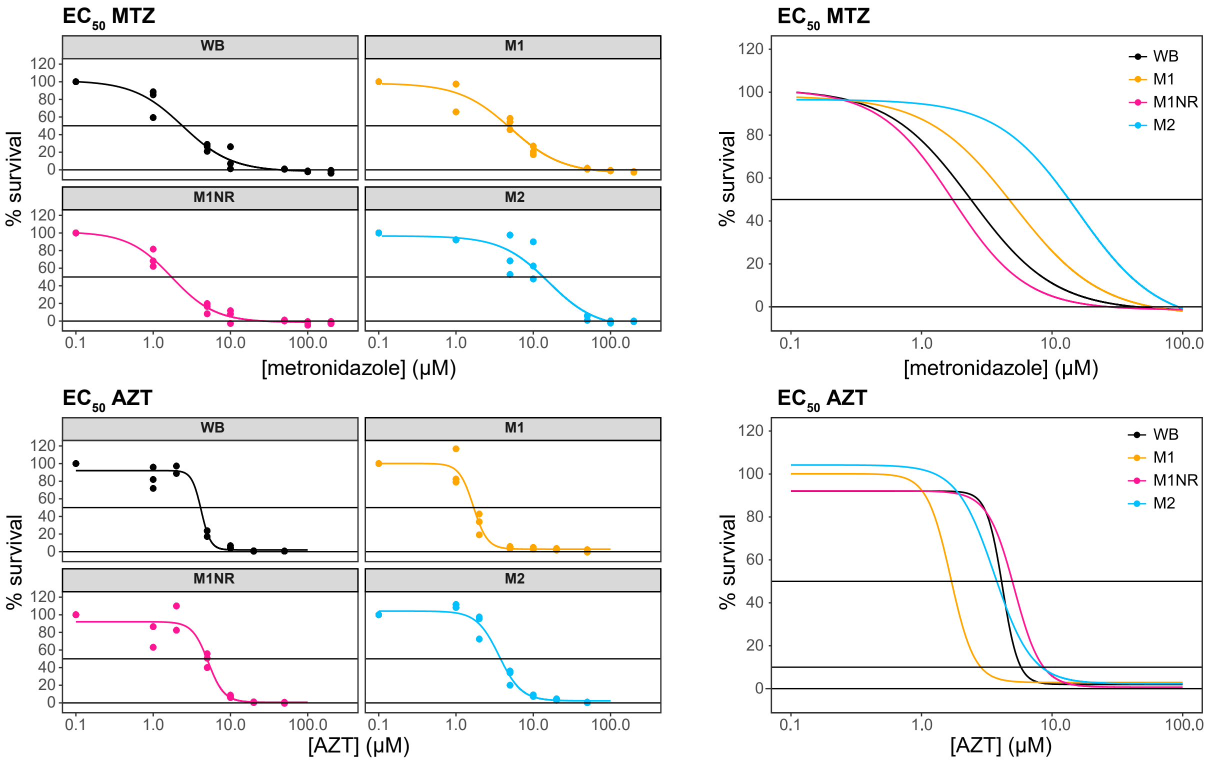

Figure S5. Inhibition of *G. intestinalis* proliferation by 72 h incubation with metronidazole (MTZ) and AZT. The treatments were performed on the WB cell line (wild type), metronidazole-resistant cells (M1 and M2), and an M1 revertant that has regained metronidazole sensitivity (M1NR). The left panels show all the individual experiments, and the right panels show a summary with each drug for all cell lines in the same plot but without the individual data points. Note that the EC_{50} values in Table 2 represent average values of the individual EC_{50} value determinations rather than from the generated average curves shown here.

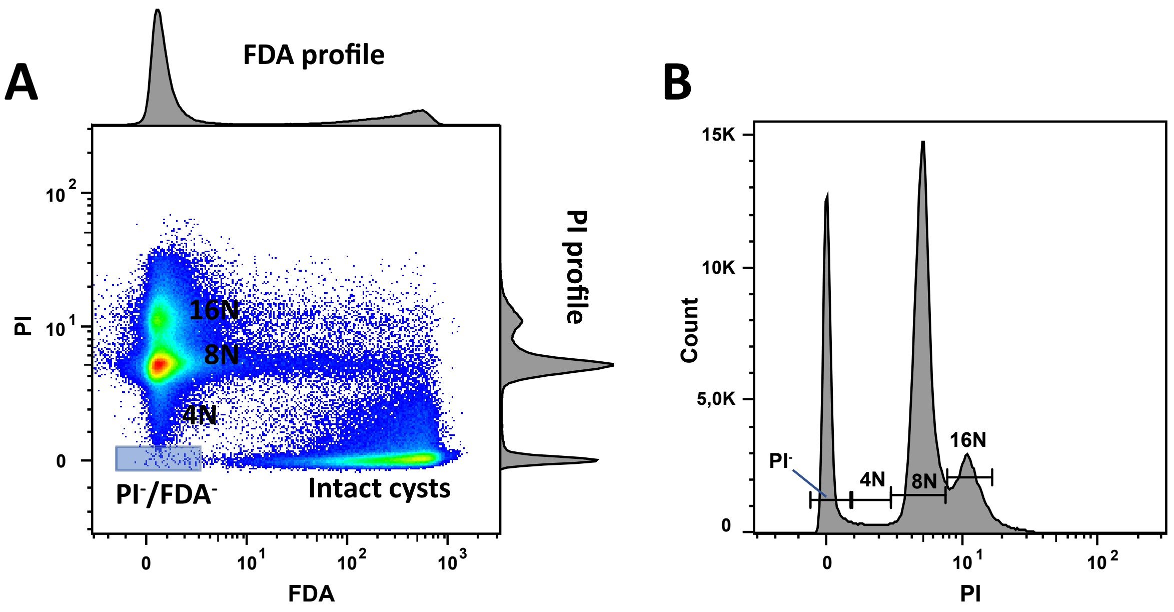

Figure S6. Illustration of the procedure for categorizing cysts based on staining with propidium iodide (PI) and fluorescein diacetate (FDA). (A) Two-dimensional plot showing the staining with both dyes. The cysts are categorized into five different groups. The first group is taken directly from the two-dimensional plot (PI^-/FDA^-) and are most likely dead because they either lack DNA or are intact but with no metabolic activity. This group is in most cases nearly empty. (B) After subtracting the PI^-/FDA^- cells, the other groups are determined from the PI profile. The remaining PI^- cells that are only labeled with FDA are counted as intact cysts because they are metabolically active (FDA measures esterase activity) and have an intact membrane that does not allow the uptake of PI. The remaining three groups are permeable for PI and show different DNA contents (4N, 8N, and 16N), but nearly all of them lack FDA staining as seen in A, indicating that they are less sturdy than the final cysts and their metabolic activity does not survive the treatments in the staining procedure. The experiments in A and B are from cells treated with only encystation growth medium and no drugs.

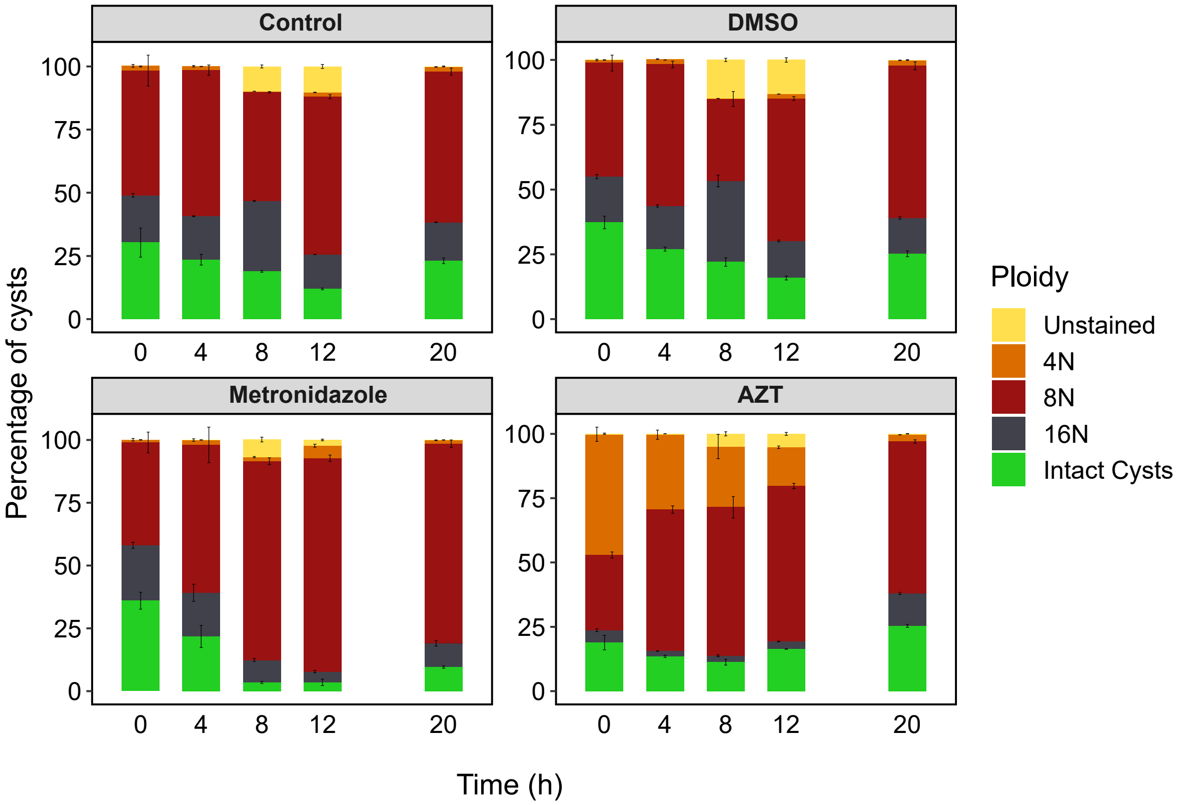

Figure S7. Effects of AZT and metronidazole on different *G. intestinalis* cyst development stages. The figure is made from the same data as Fig. 7, but the results are shown as percentages instead of absolute values. In the experiment, the cells were treated with pure encystation medium (control), DMSO, AZT, or metronidazole that were added at different time points during encystation. The cysts present after 28 h of encystation and 3 days of water treatment were categorized based on their staining with FDA (intact cysts) and PI (permeable cysts). For the permeable cysts with a leaky membrane (i.e. PI^+), different genome amplification levels could be established. Numbers are given as percentages of the total number of cysts counted at a given time point. Intact cysts are colored green, whereas PI^+ cysts are split according to their ploidy into 4N (orange), 8N (red), and 16N (black). Cysts that did not stain at all are marked in yellow. The figure shows the average of three independent experiments with standard errors.

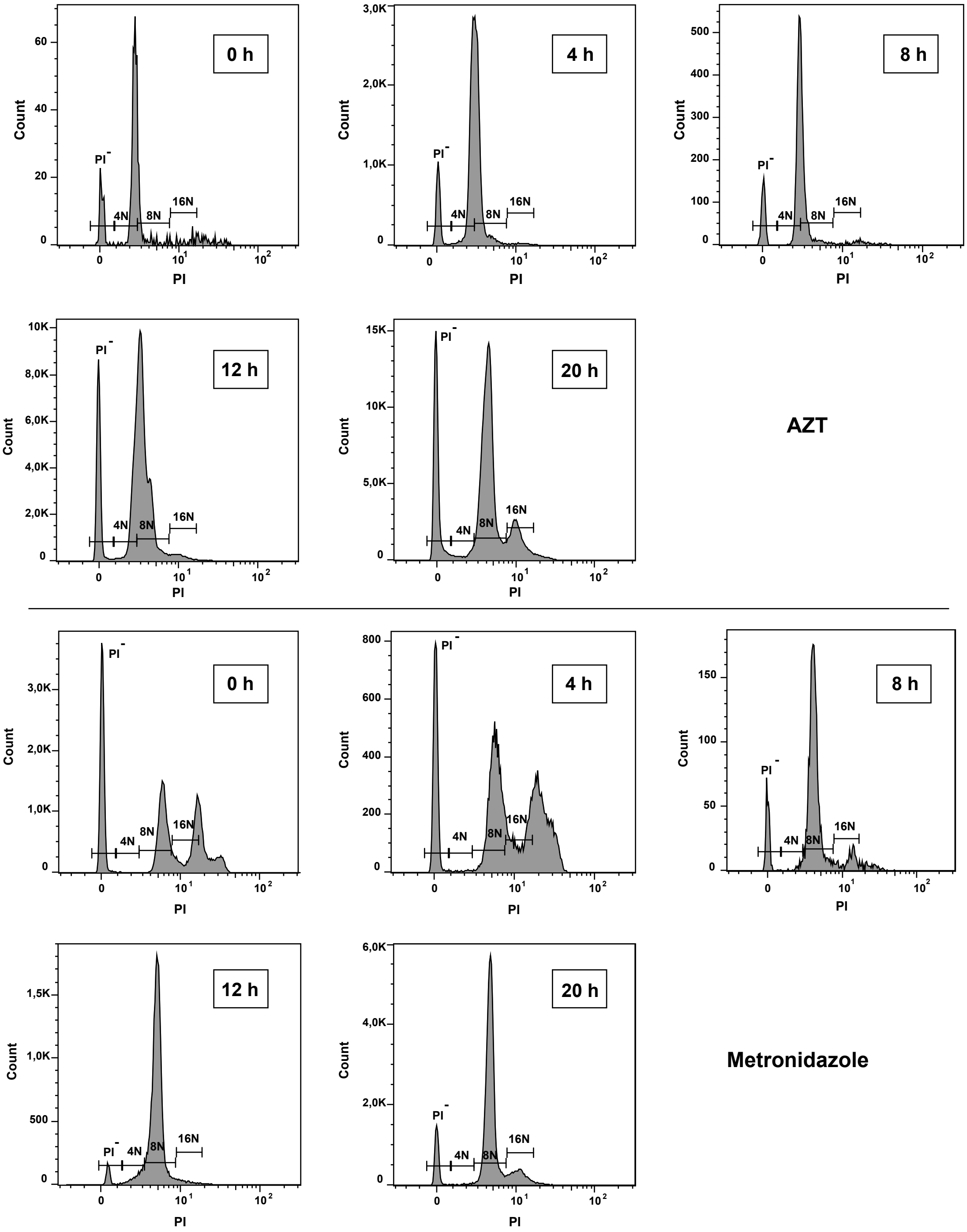

Figure S8. Effect of AZT and metronidazole on the DNA content of cysts. AZT treatment led to a clear shift of the 8N peak to the left when used at all time points except the last one at 20 h. Parts of the peak will therefore be counted as 4N in the ploidy analyses (Fig 7 and S8). A similar shift of the 8N peak did not occur when using metronidazole.

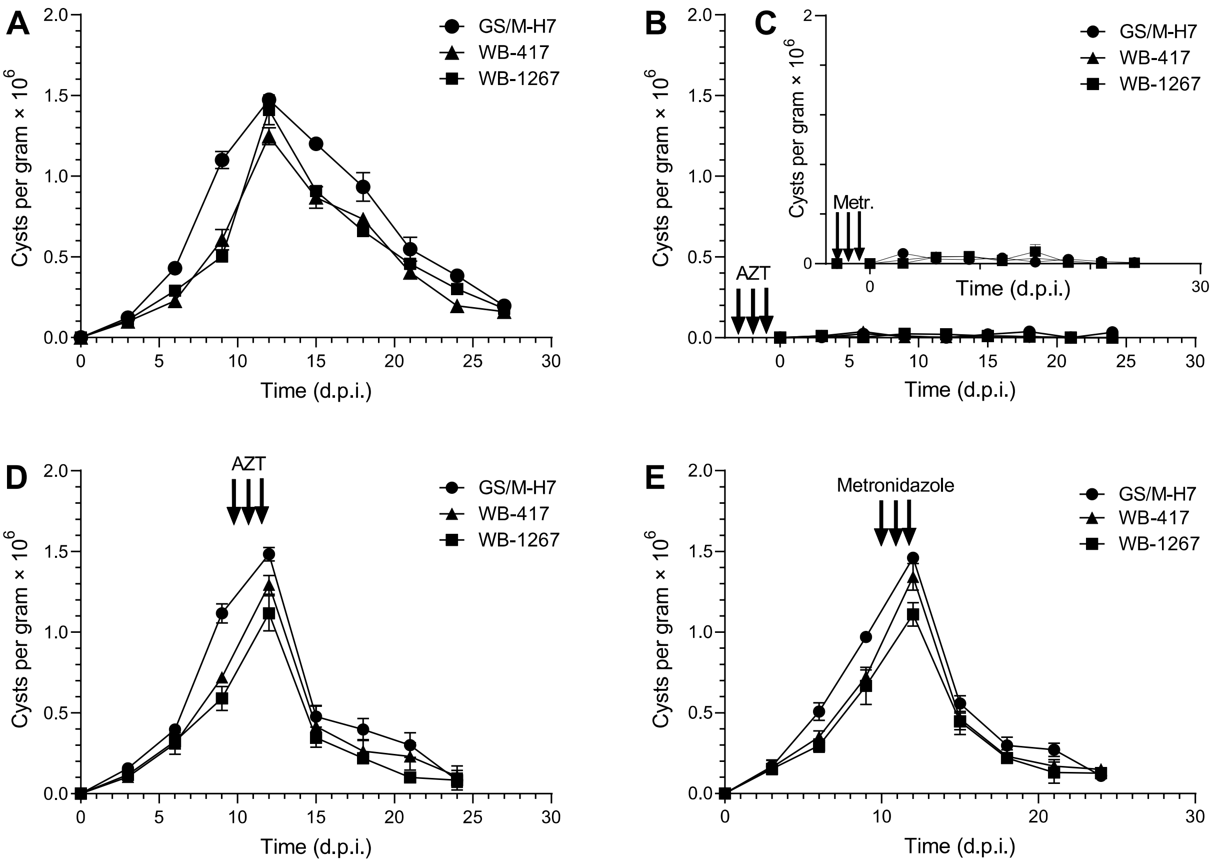

Figure S9. Effect of different treatments on the number of cysts generated in *G. intestinalis*-infected gerbils. (A) The effect of PBS (control) on the course of *G. intestinalis* infections in gerbils. The infection was monitored by counting the number of cysts per gram of stool. (B-C) The number of cysts from gerbils treated with AZT or metronidazole for 3 days prior to the infection. (D) The number of cysts from gerbils treated with AZT for 3 days starting at day 10. (E) Similar to D but with metronidazole treatment.

Table S1. Clinical signs of giardiasis in experimentally infected gerbils treated or not treated with AZT. Non-infected (PBS only) or *G. intestinalis*-infected gerbils (three different strains) were exposed to three different treatment regimens (no treatment, AZT pre-treatment, and AZT-treatment at days 10–12). This makes a total of 12 different conditions, which were performed three times (totally 36 animals). Clinical manifestations such as diarrhea (Θ), weight loss (θ), anorexia (ϕ), lethargy (‡), high temperature (■), allergies (●), or no symptom (=) were recorded. The days after infection in which the episodes occurred are shown in parentheses. No increase in body temperature or allergy was detected in any animal. In the controls, most of the evident signs of giardiasis coincided with the pick of cyst release, whereas the preventively treated animals showed no signs of giardiasis. Conversely, the animals treated with AZT between day 10 and 12 post infection ceased showing signs of the disease soon after the treatment was initiated.

Animal / Treatment	Signs (not treated)	Signs (treated for 3 days prior to infection)	Signs (treated at days 10, 11, and 12 post infection)
Gerbil 1 / PBS	=	=	=
Gerbil 2 / PBS	=	=	=
Gerbil 3 / PBS	=	=	=
Gerbil 4 / clone WB-417	Θ ϕ θ (day 7-17)	=	ϕ θ (day 8-11)
Gerbil 5 / clone WB-417	Θ ‡ θ (day 9-16)	=	‡ θ (day 8-12)
Gerbil 6 / clone WB-417	Θ ‡ θ (day 8-16)	=	Θ ‡ θ (day 6-10)
Gerbil 7 / clone WB-1267	Θ ϕ ‡ θ (day 7-15)	=	Θ ϕ θ (day 8-11)
Gerbil 8 / clone WB-1267	Θ ϕ ‡ θ (day 9-16)	=	Θ ϕ ‡ θ (day 10)
Gerbil 9 / clone WB-1267	Θ ‡ θ (day 9-17)	=	Θ ‡ θ (day 11-12)
Gerbil 10 / clone GS-H7	Θ ϕ (day 6-17)	=	Θ ϕ ‡ (day 9-11)
Gerbil 11 / clone GS-H7	Θ ϕ ‡ θ (day 8-16)	=	ϕ ‡ θ (day 9-11)
Gerbil 12 / clone GS-H7	Θ ϕ (day 6-18)	=	Θ ‡ (day 7-11)
